# Overexpression of *SLIM1* transcription factor accelerates vegetative development in *Arabidopsis thaliana*

**DOI:** 10.3389/fpls.2024.1327152

**Published:** 2024-03-20

**Authors:** Anastasia Apodiakou, Saleh Alseekh, Rainer Hoefgen, Sarah J. Whitcomb

**Affiliations:** ^1^ Department of Molecular Physiology, Max-Planck-Institute of Molecular Plant Physiology, Potsdam, Germany; ^2^ Cereal Crops Research Unit, United States Department of Agriculture - Agricultural Research Service, Madison, WI, United States

**Keywords:** Sulfur Limitation 1 (SLIM1), AT1G73730, transcription factor, leaf-size, senescence-associated genes, photosynthesis-associated genes, *Arabidopsis thaliana*, nitrate

## Abstract

The transcription factor Sulfur Limitation 1 (SLIM1) belongs to the plant-specific Ethylene Insenstive3-Like transcription factor family and is known to coordinate gene expression in response to sulfur deficiency. However, the roles of SLIM1 in nutrient-sufficient conditions have not been characterized. Employing constitutive *SLIM1* overexpression (*35S::SLIM1*) and CRISPR/Cas9 mutant plants (*slim1-cr*), we identified several distinct phenotypes in nutrient-sufficient conditions in *Arabidopsis thaliana*. Overexpression of *SLIM1* results in plants with approximately twofold greater rosette area throughout vegetative development. *35S::SLIM1* plants also bolt earlier and exhibit earlier downregulation of photosynthesis-associated genes and earlier upregulation of senescence-associated genes than Col-0 and *slim1-cr* plants. This suggests that overexpression of *SLIM1* accelerates development in *A. thaliana*. Genome-wide differential gene expression analysis relative to Col-0 at three time points with *slim1-cr* and two *35S::SLIM1* lines allowed us to identify 1,731 genes regulated directly or indirectly by *SLIM1 in vivo*.

## 1 Introduction

Plant growth is a complex process that is affected not only by genetics but also by biotic or abiotic stresses such as light, water, temperature, and nutrient availability that have an impact on hormones, cell elongation and cell division, senescence, and nutrient remobilization (Chen et al., 2017). Plant breeders seek to develop crops with increased leaf area (e.g., cassava and wheat), higher biomass (e.g., forage crops), higher yield (food crops), and early maturity (Li et al., 2017). Increasing leaf area is an important consideration in plant breeding, due to its significant impact on plant productivity. Plants with increased leaf area show improved weed suppression due to shading (Huber et al., 2021), demonstrate higher photosynthetic efficiency and CO_2_ assimilation (Faralli and Lawson, 2020), and enhanced yield (Weraduwage et al., 2015). Early maturity is a very important trait because it can help farmers avoid biotic or abiotic stress periods that tend to intensify later in the growing season, it can allow additional crop rotations per year (Mallikarjuna et al., 2019), and it can expand the productive growing region for certain crops into higher latitudes and areas with shorter growing seasons (Izawa, 2007; Wang et al., 2018).

Significant interest has surrounded transcription factors (TF) related to plant growth. For example, higher levels of the rice TF IDEAL PLANT ARCHITECTURE 1 (IPA1) decrease unproductive tillers, increase grain yield, and improve plant immunity (Wang et al., 2018). In Arabidopsis, the TFs WRKY46, WRKY54, and WRKY70 are involved in brassinosteroid-regulated growth and drought response in Arabidopsis (Chen et al., 2017). The TF bZIP30 affects the expression of genes involved in cell wall biosynthesis, cell elongation, and meristem activity in Arabidopsis (Lozano-Sotomayor et al., 2016). These examples illustrate that various TF families have been shown to affect plant growth and development through the regulation of distinct groups of genes.

During plant development, one important process for seed production is senescence. During leaf senescence, cells undergo numerous changes in cellular metabolism, such as transitioning from being a nutrient sink into being a nutrient source (Ay et al., 2009; Thomas, 2013; Heyneke et al., 2019). At this developmental stage, reproductive tissues are developing rapidly and have high nutrient needs (Fan et al., 2009), which are primarily met by remobilization of nutrients like nitrogen (N) from source organs such as leaves (Diaz et al., 2008; Thomas, 2013). Among the earliest and strongest changes in leaves during developmental senescence are chloroplast breakdown (Masclaux, 2000; Tamary et al., 2019) and decreased expression of genes involved in photosynthesis (Guo et al., 2004; Gregersen et al., 2013; Thomas, 2013). These genes are called photosynthesis-associated genes (PAGs), and their expression is negatively correlated with expression of senescence-associated genes (SAGs) (Buchanan-Wollaston et al., 2005; Lichtenthaler, 2012). SAGs are upregulated in senescing leaves (Ay et al., 2009). They are involved in processes such as degradation of chlorophyll, proteins, amino acids, nucleic acids, and autophagy (Guo et al., 2004; Lichtenthaler, 2012; Wu et al., 2012; Avila-Ospina et al., 2014). Some SAGs are ATP-Binding Cassette (ABC) transporters that facilitate nutrient transport (Wu et al., 2012). Anthocyanins tend to accumulate during leaf senescence, playing an important role in photo-protection, increasing the plant’s ability to remobilize nutrients, and protection from oxidative stress during leaf senescence (Hoch et al., 2001; Diaz et al., 2006; Liu et al., 2018).

The ETHYLENE-INSENSITIVE (EIN) family of TFs has been associated with senescence and specifically with chlorophyll degradation. ETHYLENE-INSENSITIVE2 (EIN2) controls leaf senescence through the regulation of *ETHYLENE-INSENSITIVE3* (*EIN3*), which is induced during leaf senescence (Kim et al., 2014). Via yeast 1 hybrid and chromatin immunoprecipitation-qPCR, it was shown that EIN3 binds to the promoters of two key regulators of leaf senescence, the TFs NAC DOMAIN CONTAINING PROTEIN 6 (*NAC6*) and ACTIVATED BY AP3/PI (*NAP*), and activates their transcription. EIN3 also negatively regulates *miR164*, a negative regulator of *NAC6*. These data suggest that EIN3 positively regulates leaf senescence by activating *NAC6* and *NAP* (Kim et al., 2014). The *ein3eil1* (ETHYLENE-INSENSITIVE3-LIKE 1/EIL1) double mutant exhibits delayed senescence (Li et al., 2013). This raises the question whether other members of the ETHYLENE-INSENSITIVE family could be involved in senescence.

*Sulfur Limitation 1* (*SLIM1*), an ETHYLENE INSENSITIVE 3-LIKE (EIL) family TF, is well established as a key regulatory factor involved in transcriptional responses to sulfur deficiency (−S) (Maruyama-Nakashita et al., 2006; Wawrzyńska and Sirko, 2014; Dietzen et al., 2020; Piotrowska et al., 2022; Rakpenthai et al., 2022; Apodiakou and Hoefgen, 2023). Although critical for −S responses, transcript levels of *SLIM1* are not strongly altered under –S (Maruyama-Nakashita et al., 2006; Dietzen et al., 2020; Rakpenthai et al., 2022). It has been proposed that the function of SLIM1 may be regulated by condition-dependent protein–protein interactions (PPIs) and/or post-translational modifications (Wawrzyńska and Sirko, 2014). The role of SLIM1 in sulfur-sufficient conditions is unclear. Here, we generated 35S::*SLIM1* overexpression lines and show that increased expression of *AtSLIM1* results in earlier bolting, earlier senescence, and increased rosette area relative to Col-0 and *slim1-cr*.

## 2 Materials and methods

### 2.1 Plant material

*slim1-cr* seeds were obtained from Agnieska Sirko and Anna Wawrzynska at the Institute of Biochemistry and Biophysics, Polish Academy of Sciences. Wawrzynska and colleagues used CRISPR/Cas9 to delete 1,123 bp from the AT1G73730 locus, including the previously characterized DNA binding domains of SLIM1 (Wawrzyńska and Sirko, 2016; Wawrzyńska et al., 2022) To create the 35S::SLIM1 construct, the Gateway cloning system was used (Karimi et al., 2002). The SLIM1 coding sequence was initially cloned into the pDONOR221 vector using BP clonase (Invitrogen), and then the coding sequence of SLIM1 was integrated into the pH7WG2 vector using LR clonase. The final vectors, from different colonies, were sequenced using prom35S F primer and SLIM1 R (Methods S1), and finally, the resulting positive binary plasmid was introduced into *Agrobacterium tumefaciens* (GV3101), and *Arabidopsis thaliana* Col-0 was transformed via the floral dipping method (Clough and Bent, 1998). Transgenic seeds (T1) were selected on Murashige and Skoog (MS) media containing 30 mg/L hygromycin and transferred to soil. Via TaqMan assay (Ingham et al., 2001) with an oligonucleotide hygromycin probe, the number of inserts was identified, and only seedlings with a single insert were kept for further experimental purposes. *GIGANTEA* (*GI*) (AT1G22770) was used as a single copy gene reference (Duarte et al., 2010). For the generation of empty vector (*EV*) transformed Col-0, the empty gateway PH7WG2 vector was used, following the steps mentioned above. In the experiments described in this research, *35S::SLIM1* plants were from the T2 generation carrying the *35S::SLIM1* construct. The oligonucleotide primer sequences and probes that were used for the *35S::SLIM1* construct design and verification and the conditions used for the *SLIM1* q-PCR are provided in **Supplementary Methods S1**.

### 2.2 Plant cultivation

*A. thaliana* seeds were sterilized using chlorine gas prepared by combining 100 mL of NaClO and 5 mL of HCl. The seeds were then sown on MS sterile agar medium supplemented with 1% sucrose (w/v) and were stratified at 4°C for 48 h before the plates were placed in controlled-environment chambers (CLF Plant Climatic). For the growth of *35S::SLIM1*, the MS agar plates were supplemented with 30 mg/L hygromycin, since the experiment was performed with T2 generation plants. The seeds germinated under a 16-h photoperiod with 100 µmol photons m^–2^ s^−1^ irradiance. The temperature inside the chambers was maintained at 21°C during the day and 19°C at night. Ten days after transfer into growth chambers [12 days after sowing (DAS)], the seedlings were transplanted into soil-filled round pots with a diameter of 6 cm. These pots were placed in a phytotron with controlled conditions: a photoperiod of 16 h with an irradiance of 120 µmol photons m^–2^ s^−1^, a temperature of 20°C during the day and 16°C at night, and a humidity level of 60%–75%. To minimize the potential effects of uneven lighting, the positions of the pots were regularly rearranged within the phytotron. For the molecular and metabolic experiments, T2 homozygous plants were used. TaqMan assays (Ingham et al., 2001) with oligonucleotide probes for the hygromycin gene and GI as a single copy gene were performed before 30 DAS, in order to identify homozygous plants. The stalk and the reproductive tissue were excluded from the tissue sampled, which was the rosette. The time between the stalk elimination and the rosette harvesting was approximately 4 min. The rosette samples were collected and immediately frozen in liquid nitrogen. For all time points, only rosette tissue was harvested. The samples were homogenized while frozen using a Retschmill MM400.

### 2.3 Determination of rosette area

T2 heterozygous and homozygous plants were subjected into determination of their leaf area. Photographs were taken from above once a week using a professional camera. The distance between the pots and the camera was kept consistent. Total rosette area was determined from the photos using the image analytic software, ImageJ (National Institute of Health, USA). At 44 DAS, when the stalk had visibly emerged, the stalks were carefully cut to eliminate any interference with the rosette leaf area photos.

### 2.4 Leaf microscopy

#### 2.4.1 Leaf cross-sections

Transverse strips were cut out from the middle of medium-sized leaves and then cut into smaller pieces (3–4 mm in length). The small leaf pieces were subjected to 1-h vacuum in solution A [45% ethanol, 5% glacial acetic acid, and 5% formaldehyde (37%)]. Next, samples were dehydrated by incubation in 50% ethanol for 15 min, 70% ethanol for 30 min, 80% ethanol for 1 h, and finally 90% ethanol for 1 h. Samples were left overnight at 4°C in a solution of 0.1% Eosin Y in 90% ethanol. The last dehydration step was four washes with 100% ethanol, 1 h each. Next, the samples were kept for 2 h at room temperature in solution B (equal parts 100% ethanol and Technovit 7100), then mixed well in solution B supplemented with Hardener II (15:1) for 1 min in separate tubes, and then kept at room temperature overnight to polymerize. Five-micrometer sections were prepared using Leica Rotary Microtome RM2265, placed on glass slides, and dried for 2 h at 42°C. The sections were stained with 0.05% of Toluidine blue in water and dried on a heating plate at 42°C for 10 min. The final stained sections were studied by epi-fluorescence with an Olympus BX51 microscope.

#### 2.4.2 Epithelial cells

Leaf samples for performing the epithelial cell imaging were cut out from the middle of medium-sized Arabidopsis rosette leaves of 30 and 37 DAS. The samples were treated for 1 h with 12.5% acetic acid in ethanol, then washed with 100% ethanol for 1 min, and then washed with 50% ethanol for 1 min. Multiple ddH_2_O washing steps were performed to remove ethanol from the tissue. The washed leaf tissue samples were imbibed in KOH 1 M solution where they were kept until the next day for imaging. The leaf tissue samples were studied by epi-fluorescence with an Olympus BX51 microscope. The epidermis was analyzed at 20× magnification.

### 2.5 RNA-seq

RNA-seq was performed by BGI genomics (https://www.bgi.com/global). Total RNA was extracted from approximately 100 mg of rosette tissue using the SIGMA Spectrum Plant total RNA KIT (1003037777) according to the manufacturer’s instructions with on-column DNase treatment. Following the sample preparation instructions from BGI, RNA concentration, quality, and purity were determined using a Bioanalyzer Agilent 2100. All the samples had a RIN value between 6.50 and 8.50. BGI prepared the libraries and performed the sequencing. The ribosomal RNA pool was depleted by polyA enrichment. Finally, the strand-specific libraries were sequenced with DNBseq platform (paired-end 150-bp reads with a coverage of 40 M reads per library). Clean reads were generated from raw reads by removing adaptor sequences, contamination, and low-quality reads. Specifically, SOAPnuke software was used with the following parameters: -n 0.01 -l 20 -q 0.4 –adaMR 0.25 –ada_trim –polyX 50 – minReadLen 150 (Chen et al., 2018). The quality of clean reads was confirmed using FastQC v0.12.1 prior to downstream analysis. The clean reads were aligned to the Arabidopsis genome (NCBI assembly TAIR10) using CLC genomics v23.0.4 (Keohavong and Grant, 2004) with default settings. The genome annotations are based on Araport11. Read counts per gene were normalized to account for differences in sequencing depth and RNA composition among samples using the median of ratios method in the DESeq2 v1.40.2 R package (Love et al., 2014). Differential expression analysis was performed with DESeq2 using Wald tests with Empirical Bayes-based adaptive shrinkage of exaggerated log2FoldChanges using the ashr v2.2-54 R package (Stephens et al., 2022) and *p*-value adjustment using the IHW v1.28.0 R package (Ignatiadis et al., 2016; Ignatiadis and Huber, 2021), which implements independent hypothesis weighting. To annotate genes with Gene Ontology terms and KEGG pathways, BiomaRt v2.56.1 (Durinck et al., 2005, 2009) and KEGGREST v1.40.0 R (Tenenbaum and Maintainer, 2023) packages were used to access (June 2023) the EnsemblPlants (https://plants.ensembl.org) and Kyoto Encyclopedia of Genes and Genomes (https://www.kegg.jp) databases, respectively. Over-representation analysis was performed with the clusterProfiler v4.0 (T. Wu et al., 2021) R package. Data visualizations were prepared with ggplot2 v3.4.2 (Wickham, 2016), UpSetR v1.4.0 (Conway et al., 2017; Gehlenborg, 2019), ComplexHeatmap v2.16.0 (Gu et al., 2016), and circlize v0.4.15 (Gu et al., 2014) R packages.

### 2.6 *In silico* analysis of *cis*-regulatory elements and motifs

Patmatch (https://www.arabidopsis.org/cgi-bin/patmatch/nph-patmatch.pl) (Yan et al., 2005) was used to determine the presence of *cis*-regulatory elements and motifs of interest in the promoters of putative SLIM1-target genes, such as the genes bound by SLIM1 in DAP-seq, the class 1 SLIM1-regulated gene set, and the 12 genes that were both bound by SLIM1 in DAP-seq and met the class 1 criteria in our dataset (**Supplementary Table S4**). We used default settings with the exception of the search window, which was −1,000 bp upstream of the transcription start site.

### 2.7 Chlorophyll and anthocyanin quantification

Homogenized tissue (20 mg) was dissolved in 300 μL of ice-cold 95% (v/v) ethanol, briefly vortexed, and kept on ice until all the samples were processed. To remove debris, the samples were centrifuged at 14,000 × *g* for 5 min. The supernatant was carefully transferred to a new tube, which was then placed on ice and covered with foil to prevent chlorophyll degradation. For absorbance determination, 100 μL of the supernatant was brought to 200 μL with 95% (v/v) ethanol and put in a flat-bottom 96-well plate. Absorbance measurements were taken at wavelengths of 664.1 nm, 648.6 nm, 470 nm, and 750 nm (blank). Chl a and Chl b concentrations were calculated using the following formulas (Lichtenthaler and Buschmann, 2001), and calculated concentrations were normalized to the fresh weight (FW) of the extracted sample.

Chl a (μg/mL) = 13.36×A_664.1_ – 5.19×A_648.6_
Chl b (μg/mL) = 27.43×A_648.6_ – 8.12×A_664.1_

For anthocyanin content measurements, 90 μL of the chlorophyll-containing supernatant was mixed with 10 μL of 1 M HCl, and the absorbance was measured at 520 nm and 750 nm (blank) (Lee et al., 2008). Absolute absorbance was normalized to the FW of the extracted sample.

### 2.8 Metabolite and ion determination

Metabolites were extracted as previously described by Lisec et al. (2006) with few modifications (Guo, 2018). Briefly, 25 mg of homogenized tissue was mixed with ice-cold CHCl_3_/CH_3_OH (3:7, v/v) buffer supplemented with Isovitexin and ^13^C6-sorbitol for internal standards. The samples were placed at −20°C for 2 h and vortexed briefly every 30 min. Ice-cold water was added, and samples were vortexed until the two phases were dispersed. Samples were centrifuged at 14,000 × *g* for 10 min at 4°C. The upper, polar phase was transferred to a new tube and desired volumes were further aliquoted for LC-MS (200 μL) and ion chromatography. The non-polar phase was also transferred to a new tube. The polar phase aliquots, non-polar phase, and insoluble material were evaporated using a centrifugal vacuum dryer at 30°C for 5 h. LC-MS was performed essentially as previously described (Perez de Souza et al., 2019, 2021). For ion content determination, the evaporated polar phase was dissolved in high-purity H_2_O and analyzed by Dionex ICS-3000 system using a KOH gradient (6–55 mM, 0.25 mL/min flow rate) for anion analysis with 17 min duration of the gradient. For cation analysis, methanesulfonic acid gradient (60–100 mM, 0.3 mL/min flow rate) was used with 20 min duration of gradient. The final ion concentration in the samples was calculated based on the known concentration of the following standards: CaCl_2_, NaCl, KH_2_PO_4_, (NH4)_2_SO_4_, MgCl_2_, MgSO_4_, and KNO_3_. The standards had a concentration range between 2 and 200 μM.

### 2.9 Thiol extraction and derivatization

Extraction was performed as previously described (Watanabe et al., 2008; Guo, 2018). Briefly, 25 mg of homogenized tissue was mixed with 0.1 M HCl, and samples underwent a second round of homogenization using a Retschmill to achieve optimal cell lysis. For the reduction step with 1.58 M *N*-ethylmorpholine, the extract was supplemented with 25 μM *N*-acetyl-Cys, as the internal standard. The reaction was allowed to react with 25 mM phosphine for 20 min at 37°C. For the labeling step, the reduced sample was allowed to react with 30 mM monobromobimane (mBrB) for 20 min at 37°C in the dark. The labeling reaction was terminated by the addition of acetic acid, and the resulting solution was then subjected to HPLC analysis. HPLC was performed as previously described (Watanabe et al., 2008).

## 3 Results

### 3.1 Plants overexpressing *SLIM1* transcription factor have larger rosettes during the vegetative growth phase

**Figure 1:**
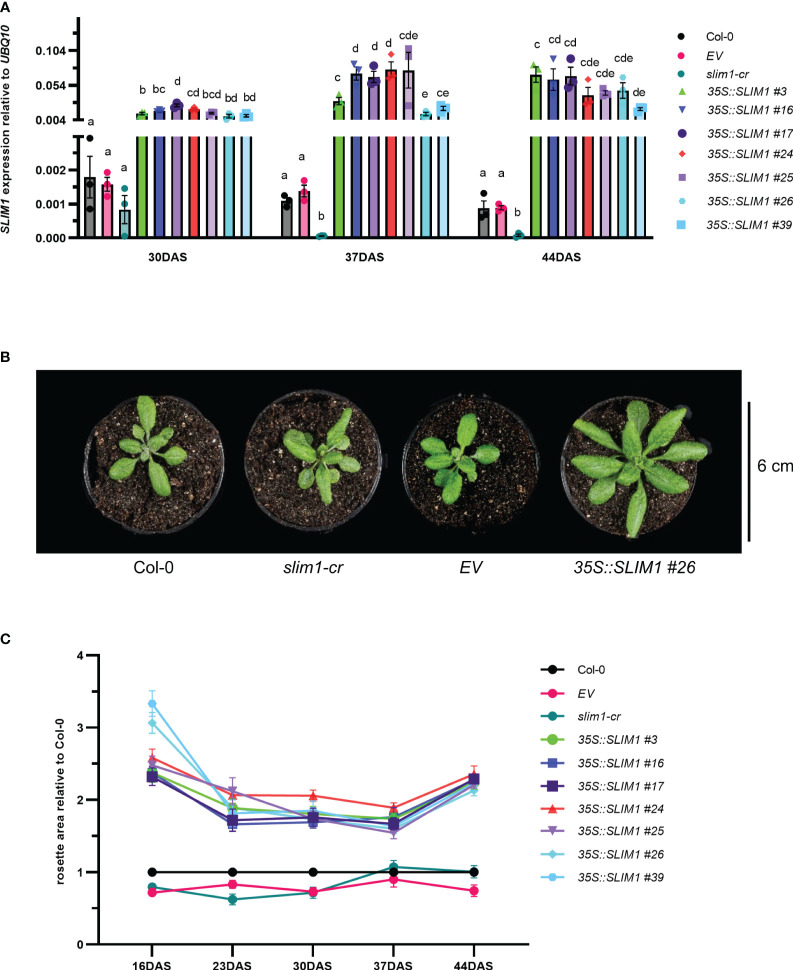
Effect of SLIM1 overexpression on growth. **(A)** *SLIM1* transcript level relative to *UBQ10* determined by qPCR. Bar height corresponds to mean of three biological replicates, and error bars represent the standard error of the mean (SEM). Statistical significance was assessed using *t*-tests within each time point; *p*-values were adjusted by the Benjamini–Yekutieli method. Compact letter display (CLD) identifies lines that are statistically different (adjusted *p* ≤ 0.05) from each other at each time point. **(B)** Photo of representative rosettes at 30 DAS. **(C)** Rosette area relative to Col-0 at each time point. Points represent the mean of 10–20 biological replicates, and error bars show the SEM. See **Supplementary Table S1** for *t*-test results.

T2 *35S::SLIM1* seeds from seven independent lines were germinated on agar medium, and hygromycin-resistant seedlings were transferred to soil. *SLIM1* expression at 30, 37, and 44 DAS was assessed by qPCR (Methods S1). *AtSLIM1* transcript levels were significantly higher in the *35S::SLIM1* lines than in Col-0, *EV* (empty vector control), and *slim1-cr* (**Figure 1A**). In the *slim1-cr* line, the *slim1* locus has a 1,123-bp deletion including the regions coding for the DNA binding domains, but the 3’-portion of the locus remains intact. However, transcripts from the remaining portion of the locus in *slim1-cr* were found to be reduced to approximately 20% of the level in Col-0, indicating that *slim1-cr* is likely a loss of function line (Wawrzyńska et al., 2022).

It was readily apparent by visual inspection that *35S::SLIM1* plants have larger rosettes (**Figure 1B**, **Supplementary Figure S1**), and quantification of rosette area in the seven independent *35S::SLIM1* lines showed consistently 1.5- to 3-fold larger rosettes than in controls throughout the vegetative growth phase (**Figure 1C**). Furthermore, from 16 DAS to 37 DAS, rosette biomass was consistently two- to sixfold greater in the seven *35S::SLIM1* lines (**Supplementary Figure S2A**). Mesophyll cells account for most of the cellular biomass in Arabidopsis leaves, so we investigated whether the number or size of mesophyll cells differed among the genotypes. Histological cross-sections from Arabidopsis mature leaves at 35 DAS were treated with Toluidine blue to stain cell walls and were visualized by epi-fluorescence (**Supplementary Figure S2B**). The number of mesophyll cells per unit area was not altered in *35S::SLIM1* or *slim1-cr* relative to Col-0 (**Supplementary Figure S2C**). Given the larger rosette phenotype in *35S::SLIM1*, the distribution of epithelial cell sizes in each genotype at 30 and 37 DAS was determined (**Supplementary Figures S2D, E**). The size of epithelial cells was not significantly different in *35S::SLIM1* or *slim1-cr* relative to Col-0 and *EV* at either time point (**Supplementary Figure S2E**).

### 3.2 Plants overexpressing *SLIM1* accumulate anthocyanins and the flavonoid pathway is induced

**Figure 2:**
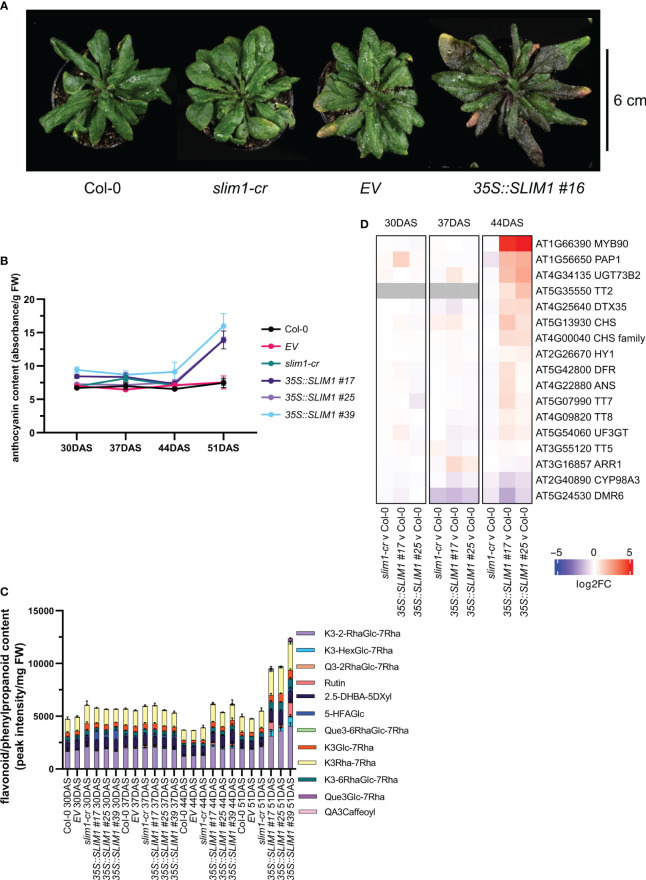
Late effect of SLIM1 overexpression on anthocyanin content. **(A)** Photo of representative rosettes at 51 DAS. **(B)** Anthocyanin content normalized to fresh weight (FW). Points represent the mean of three biological replicates, and error bars show the SEM. See **Supplementary Table S1** for *t*-test results. **(C)** Sample FW normalized content of flavonoid and phenylpropanoid compounds identified by LC-MS. The simplified codes for flavonoids correspond to the following compounds: kaempferol-3-*O*-(2”-*O*-rhamnosyl)glucoside-7-*O*-rhamnoside: K3-2-RhaGlc-7Rha; kaempferol-3-*O*-(-*O*-hexosyl)glucoside-7-*O*-rhamnoside: K3-HexGlc-7Rha; keampferol-3-*O*-rhamnoside-7-*O*-rhamnoside: K3Rha-7Rha; quercetin-3-*O*-(2”-*O*-rhamnosyl)glucoside-7-*O*-rhamnoside: Q3-2RhaGlc-7Rha; Rutin: Rutin; 2,5-dihydroxybenzoic acid 5-*O*-D-xyloside: 2.5-DHBA-5DXyl; 5-hydroxyferulic acid glucoside: 5-HFAGlc; kaempferol-3-*O*-6-*O*-(rhamnosyl)glucoside-7-*O*-rhamnoside: K3-6RhaGlc-7Rha; kaempferol-3-*O*-glucoside-7-*O*-rhamnoside: K3Glc-7Rha; quercetin-3-*O*-6-*O*-(rhamnosyl)glucoside-7-*O*-rhamnoside: Que3-6RhaGlc-7Rha; quercetin-3-*O*-glucoside-7-*O*-rhamnoside: Que3Glc-7Rha; quinic acid, 3-caffeoyl: QA3Caffeoyl. Bar height of each compound corresponds to the mean of three biological replicates, and error bars show the SEM. See **Supplementary Table S1** for *t*-test results. **(D)** Heatmap representation of gene expression differences determined by RNA-seq. Genes shown include *MYB75* and *MYB90* and those genes annotated to gene ontology terms related to anthocyanin and flavonoid pathways (GO:0009813 and GO:0031537) that are expressed in rosette tissue based on published literature and information in TAIR database. Color corresponds to log2 of the fold change (log2FC) relative to Col-0 at each time point determined from three biological replicates. Wald test results for genes shown can be found in **Supplementary Table S1**.

When extending the growth phase to 51 DAS, plants overexpressing *SLIM1* display a purple/pink pigmented phenotype (**Figure 2A**). Anthocyanins are known to contribute to purple/pink tissue appearance, and so anthocyanin content was measured in the rosettes. Total anthocyanin content increased significantly in *35S::SLIM1* plants between 44 DAS and 51 DAS (**Figure 2B**), which is consistent with the timing of the purple pigmented phenotype in the leaves of *35S::SLIM1* at 51 DAS (**Figure 2A**). Since anthocyanins are one subgroup of flavonoids (Tohge et al., 2017; Liu et al., 2018), we determined flavonoid contents. Similar to the anthocyanin profile, at late time points, plants overexpressing *SLIM1* displayed an accumulation of various flavonoids, such as quercetins and kaempferols, compared to Col-0 and *slim1-cr* (**Figure 2C**). The differential accumulation of flavonoids is evident at 44 DAS, and the relative accumulation was even greater at 51 DAS.

To investigate the gene expression changes that may underlie the differential accumulation of flavonoids including anthocyanins late in development, we performed transcriptome profiling by RNA-seq on two independent *35S::SLIM1* lines, *slim1-cr*, *EV* and Col-0. In these data, we analyzed the expression of genes annotated to GO terms related to flavonoid/anthocyanin pathways (GO:0009813 and GO:0031537) and TFs PAP1/*MYB75* (AT1G56650) and PAP2/*MYB90* (AT1G66390) known to regulate genes in the flavonoid/anthocyanin pathway (Borevitz et al., 2000; Tohge et al., 2005; Zuluaga et al., 2008). At 30 and 37 DAS, expression of these genes in the *SLIM1* mutants was similar to Col-0. However, at 44 DAS, transcripts of the TFs *PAP1*/*MYB75* and *PAP2*/*MYB90* were found to be approximately 4-fold and 32-fold higher in *35S::SLIM1* lines than in Col-0 (**Figure 2D**). Genes coding for enzymes such as *UGT73B2* (AT4G34135) and *CHS* (AT5G13930), are two- to fourfold induced in both *35S::SLIM1* lines relative to Col-0 (**Figure 2D**). Furthermore, the TF *TT2* (AT5G35550) that regulates proanthocyanidin synthesis (Y. Liu et al., 2015; Zhu et al., 2015) was found to be upregulated in *35S::SLIM1* at 44 DAS (**Figure 2D**).

### 3.3 Overexpression of *SLIM1* TF promotes developmental senescence

**Figure 3:**
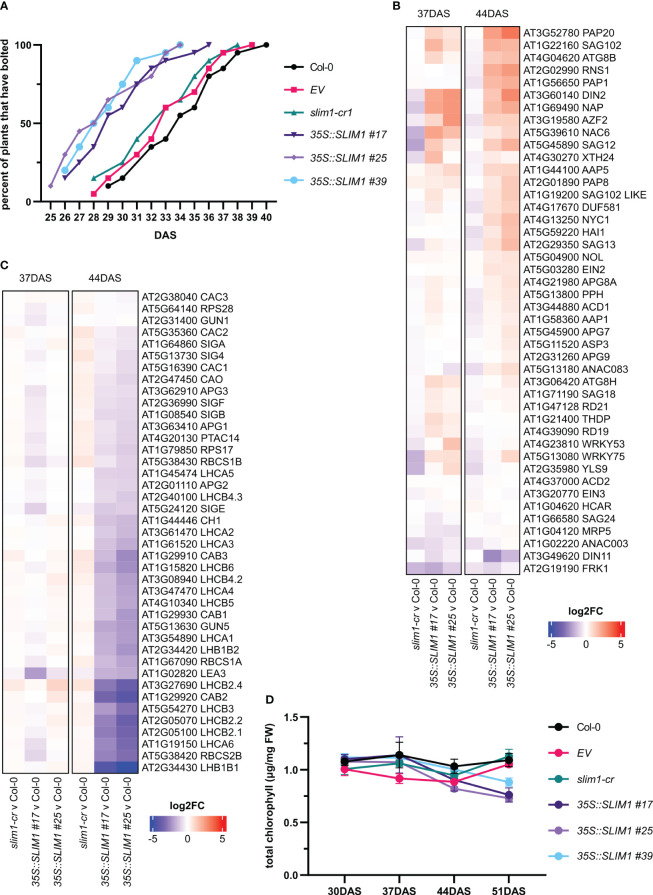
Development is accelerated in *35S::SLIM1* lines. **(A)** Cumulative percent of plants that have visibly bolted among 20 plants of each line as a function of DAS. Plants were considered to have bolted from the first day that the shoot apical meristem was visible. See **Supplementary Table S1** for *t*-test results. **(B)** Heatmap representation of gene expression differences determined by RNA-seq. Genes shown are SAGs collected from published literature (Balazadeh et al., 2008; Li et al., 2012; Kim et al., 2014; Sakuraba et al., 2014; Gao et al., 2016; Hsu et al., 2022). **(C)** Heatmap representation of gene expression differences determined by RNA-seq. Genes shown are PAGs collected from published literature (Sawchuk et al., 2008; Horie et al., 2009; Gao et al., 2012; Motohashi et al., 2012; Yu et al., 2017; Liang et al., 2019). **(B, C)** Heatmap color corresponds to log2 of the fold change (log2FC) relative to Col-0 at each time point determined from three biological replicates. Wald test results for genes shown can be found in **Supplementary Table S1**. **(D)** Total chlorophyll content normalized to sample FW. Points represent the mean of three biological replicates, and error bars show the SEM. See **Supplementary Table S1** for *t*-test results.

All genotypes started to visibly bolt at the 10-leaf stage, but the mean bolting day of *35S::SLIM1 plants* was 29 DAS, while for Col-0, *EV*, and *slim1-cr*, it was 33 DAS (**Figure 3A**). Earlier bolting together in 35S::SLIM1 plants with accumulation of flavonoids and chlorosis at the leaf tips, which is typical for onset of senescence (Watanabe et al., 2013), observed at 51 DAS in the *35S::SLIM1* genotype (**Figures 2A–C**), indicates a faster development that may lead to earlier senescence in *35S::SLIM1* compared to Col-0, *EV*, and *slim1-cr*. To assess this hypothesis, at 37 and 44 DAS, the expression of genes known to be differentially regulated during senescence, such as SAGs and PAGs, were analyzed. Various SAGs were strongly upregulated in *35S::SLIM1* compared to the controls (**Figure 3B**, **Supplementary Table S1**). Genes such as *SAG12* (AT5G45890) and *SAG18* (AT1G71190) (Balazadeh et al., 2008; Gao et al., 2016; Hsu et al., 2022), *NAC6* (AT5G39610) and *NAP* (AT1G69490) (Li et al., 2013; Kim et al., 2014), and autophagy genes including *APG8A*/*ATG8A* (AT4G21980), *ATG8B* (AT4G04620) and *ATG8H* (AT3G06420) (Li et al., 2012; Sakuraba et al., 2014) are upregulated in *35S::SLIM1*. Genes involved in chlorophyll degradation such as *NYC1* (AT4G13250) and *PPH* (AT5G13800) (Li et al., 2012) were found to be induced in *35S::SLIM1* and either downregulated or unaltered in *slim1-cr* (**Figure 3B**).

In contrast, most PAGs are at least twofold downregulated in both *35S::SLIM1* lines relative to Col-0 at 44 DAS (**Figure 3C**, **Table 1**, **Supplementary Table S1**). *LHC* family (Sawchuk et al., 2008; Horie et al., 2009) and *CAC* genes that code for chloroplast localized proteins (Yu et al., 2017) are downregulated in *35S::SLIM1*. *LEA3* (AT1G02820), which enhances photosynthetic efficiency by reducing ROS (Liang et al., 2019) and *APG* genes, whose mutants result in albino plants (Motohashi et al., 2012) and are downregulated in *35S::SLIM1* (**Figure 3C**). Another important PAG gene is *PTAC14* (AT4G20130), which regulates chloroplast development (Gao et al., 2012), and is also downregulated in *35S::SLIM1.* Since PAGs are involved in photosynthesis and in chlorophyll biosynthesis, we measured the chlorophyll content in the plants. Total chlorophyll content (**Figure 3D**) decreases steadily between 37 and 51 DAS in *35S::SLIM1* lines, while in Col-0, *EV*, and *slim1-cr*, total chlorophyll content remains similar to earlier time points (**Figure 3D**).

**Table 1:** Differential expression summary table for SAG and PAG gene sets.

Gene set^†^	Set size^‡^	Contrast	Time point	DE^§^	DE and up^¶^	DE and down^††^
SAG	1,385	*slim1-cr* v Col-0	37 DAS	268	52	216
SAG	1,385	*35S::SLIM1 #17* v Col-0	37 DAS	625	425	200
SAG	1,385	*35S::SLIM1 #25* v Col-0	37 DAS	454	298	156
SAG	1,396	*slim1-cr* v Col-0	44 DAS	352	125	227
SAG	1,396	*35S::SLIM1 #17* v Col-0	44 DAS	619	329	290
SAG	1,396	*35S::SLIM1 #25* v Col-0	44 DAS	671	386	285
PAG	1,279	*slim1-cr* v Col-0	37 DAS	157	102	55
PAG	1,279	*35S::SLIM1 #17* v Col-0	37 DAS	646	68	578
PAG	1,279	*35S::SLIM1 #25* v Col-0	37 DAS	171	82	89
PAG	1,280	*slim1-cr* v Col-0	44 DAS	567	500	67
PAG	1,280	*35S::SLIM1 #17* v Col-0	44 DAS	815	0	815
PAG	1,280	*35S::SLIM1 #25* v Col-0	44 DAS	896	0	896

† Senescence-associated genes (SAG) are annotated to one or more of the following GO terms: GO:0006914, GO:0006561, GO:0016070, GO:0009850, GO:0016042, GO:0016209, GO:0010150, GO:0009737, GO:0009813, GO:0010498, GO:0006511, GO:0015996, GO:0006401, GO:0090304, and GO:0031542. Photosynthesis-associated genes (PAG) are annotated to one or more of the following GO terms: GO:0015995, GO:0009534, GO:0009507, GO:0009523, GO:0009522, and GO:0015979.

‡ Number of genes in set that were tested for differential expression by Wald’s test.

§ Number of differentially expressed (DE) genes: adjusted *p*-value < 0.05.

¶ Number of genes that were both differentially expressed and upregulated: adjusted *p*-value < 0.05 and log2FoldChange > 0.

†† Number of genes that were both differentially expressed and downregulated: adjusted *p*-value < 0.05 and log2FoldChange < 0.

**Figure 4:**
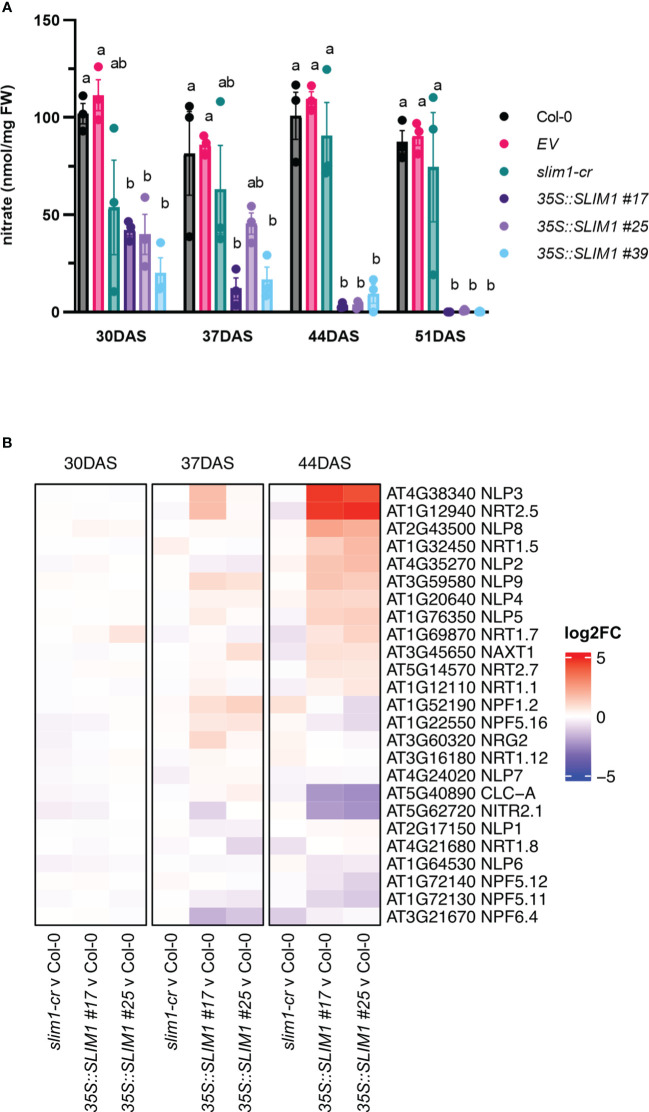
Rosette nitrate content is reduced in *35S::SLIM1* lines. **(A)** Rosette nitrate content normalized to sample FW. Bar height corresponds to mean of three biological replicates, and error bars represent the standard error of the mean (SEM). Statistical significance was assessed using *t*-tests within each time point; *p*-values were adjusted by the Benjamini–Yekutieli method. Compact letter display (CLD) identifies lines that are statistically different (adjusted *p* ≤ 0.05) from each other at each time point. **(B)** Heatmap representation of gene expression differences determined by RNA-seq. Genes shown include genes annotated to GO term nitrate transmembrane transport (GO: 0015706). Heatmap color corresponds to log2 of the fold change (log2FC) relative to Col-0 at each time point determined from three biological replicates. Wald test results for genes shown can be found in **Supplementary Table S1**.

Since nitrogen remobilization is tightly linked to senescence in Arabidopsis (Heyneke et al., 2017, 2019; Masclaux-Daubresse et al., 2010), rosette nitrate (NO_3_^−^) levels were determined. NO_3_^−^ contents are lower in the *35S::SLIM1* lines from as early as 30 DAS, and the plants undergo further NO_3_^−^ reduction as they age (**Figure 4A**). In contrast, over the same time course, very little change in NO_3_^−^ was observed in Col-0, *EV*, and *slim1-cr*. Nitrate transmembrane transport genes were checked for differential expression. The nitrate transporter *NRT2;5* (AT1G12940) known to be induced under low NO_3_^−^ and under N-starvation conditions (Liu et al., 2020), is highly overexpressed in *35S::SLIM1* (**Figure 4B**) at 44 DAS. At this time point, NO_3_^−^ content in *35S::SLIM1* is only 5% of the levels at 30 DAS. Additionally, *NRT1;5* (AT1G32450), an important long-distance root-to-shoot transporter (Chen et al., 2021), and *NRT1;7* (AT1G69870), a source-to-sink transporter (Fan et al., 2009), are induced in *35S::SLIM1* at 44 DAS relative to Col-0 (**Figure 4B**). The chloride channel *CLC-A* (AT5G40890) has a main role in the control of the NO_3_^−^ status in Arabidopsis and is repressed under NO_3_^−^-deficient conditions (Geelen et al., 2000). *CLC-A* expression is strongly downregulated in *35S::SLIM1* at 44 DAS, a time point at which the NO_3_^−^ content is particularly low in these lines (**Figures 4A, B**). Moreover, six NO_3_^−^-responding TFs, NIN-like RWP-RK domain-containing proteins (*NLP*) (Luo et al., 2021), are upregulated in the *35S::SLIM1* lines compared to Col-0 and *slim1-cr* at 44 DAS (**Figure 4B**).

### 3.4 Effects of *SLIM1* expression on transcriptome in adult developmental stages

Given the multiple developmental phenotypes we observed in the *35S::SLIM1* lines, we wanted to assess the impact of *SLIM1* expression on the transcriptome more generally (**Supplementary Data Sheet S3**). Using an adjusted *p*-value cutoff < 0.05, 9,228 and 10,198 genes were found to be differentially expressed (DE) at 44 DAS in *35S::SLIM1 #17* and *#25*, respectively, compared to Col-0, while 5,002 genes are DE in *slim1-cr* compared to Col-0 at 44 DAS (**Supplementary Figure S3**). At both 37 DAS and 44 DAS, the number of differentially expressed genes (DEG) is approximately two- to threefold higher in *35S::SLIM1* lines than in *slim1-cr*. Additionally, the number of DEGs in the *EV* line are far fewer than in the *SLIM1* mutants (91 in *EV* and an average of 2,292 in the *35S::SLIM1* lines at the same time point), which implies that the transcriptional effect of the vector itself on *35S::SLIM1* plants is not significant.

Among the DEGs at each time point, we wanted to identify those genes with the strongest transcriptome evidence of being regulated by SLIM1, either directly or indirectly. For this purpose, we defined a set of so-called class 1 SLIM1-regulated genes. Class 1 genes at each time point are characterized as being DE in *35S::SLIM1 #17*, *35S::SLIM1 #25*, and *slim1-cr* and as having consistent behavior in both *35S::SLIM1* and opposite behavior in *slim1-cr*. Class 1 SLIM1 positively regulated genes (*35S::SLIM1* UP & *slim1-cr* DOWN) are DE and upregulated (log2FC > 0) in both *35S::SLIM1* lines, and in *slim1-cr*, they are DE and downregulated (log2FC < 0). Similarly, Class 1 SLIM1 negatively regulated genes (*35S::SLIM1* DOWN & *slim1-cr* UP) are DE and downregulated in both *35S::SLIM1* lines, and in the *slim1-cr* line, they are DE and upregulated (**Supplementary Table S2**).

**Figure 5:**
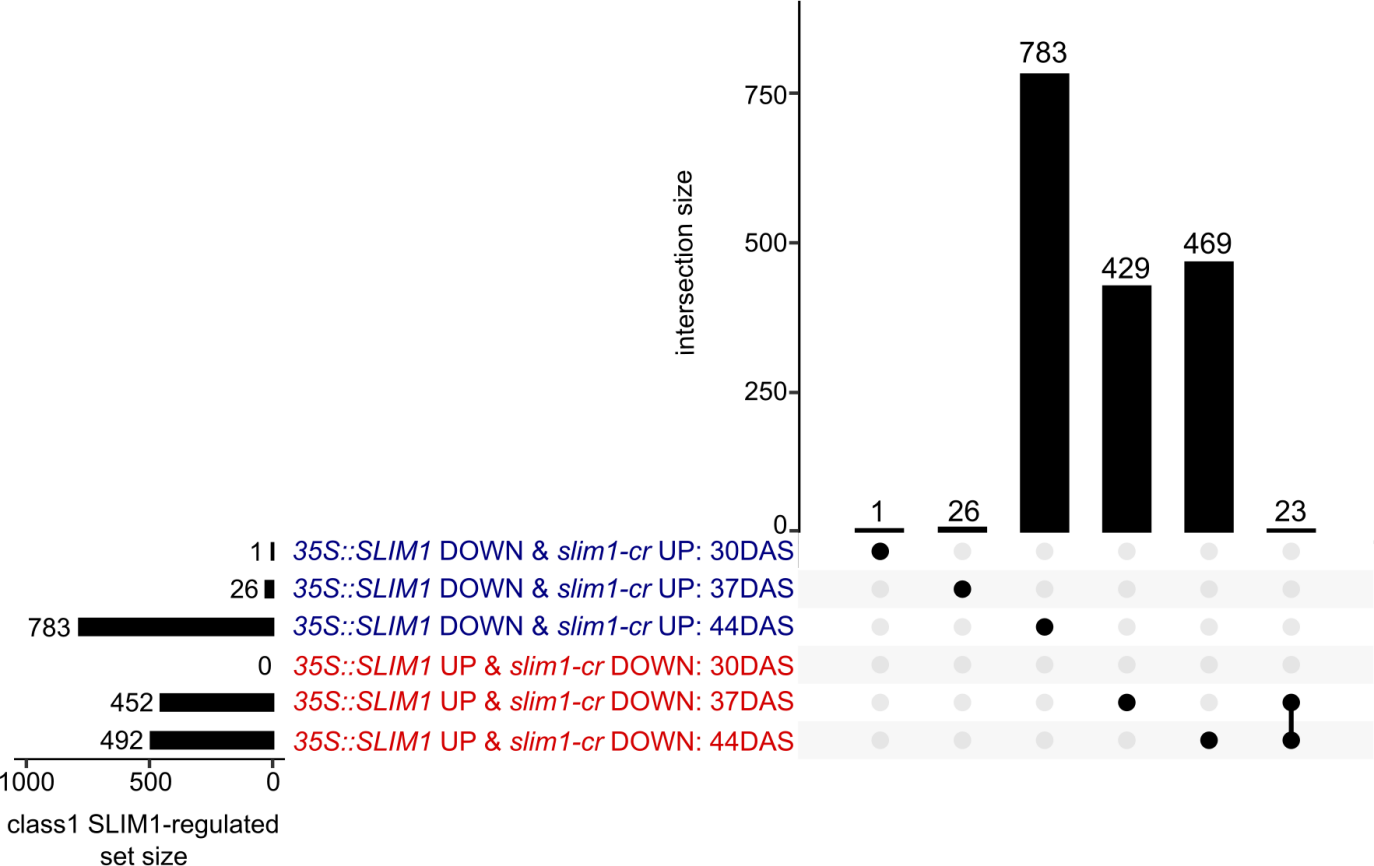
Class 1 SLIM1-regulated gene set intersection analysis. UpSet plot of class 1 SLIM1-regulated genes at each time point. Class 1 genes are defined as being either upregulated (*p*_adj_ < 0.05 and log2FC > 0) in both *35S::SLIM1* lines and downregulated (*p*_adj_ < 0.05 and log2FC < 0) in *slim1-cr*, *35S::SLIM1* UP & *slim1-cr* DOWN), or downregulated (*p*_adj_ < 0.05 and log2FC < 0) in both *35S::SLIM1OX* lines and upregulated (*p*_adj_ < 0.05 and log2FC > 0) in *slim1-cr*, (*35S::SLIM1* DOWN & *slim1-cr* UP). Wald test results for class 1 genes shown can be found in **Supplementary Table S1**. Genes in each set and intersection can be found in **Supplementary Table S2**.

Based on these criteria, a total of 1,731 genes were identified as class 1 SLIM1-regulated genes. At 37 DAS, 478 class 1 genes were identified, 452 *35S::SLIM1* UP & *slim1-cr* DOWN and 26 *35S::SLIM1* DOWN & *slim1-cr* UP (**Figure 5**). At 44 DAS, 1,275 class 1 genes were identified, 492 *35S::SLIM1* UP & *slim1-cr* DOWN and 783 *35S::SLIM1* DOWN & *slim1-cr* UP. Among the 1,731 class 1 genes, 23 met the criteria at both 37 and 44 DAS, and all 23 had expression behavior consistent with their being positively regulated by SLIM1 (**Figure 5**).

**Figure 6:**
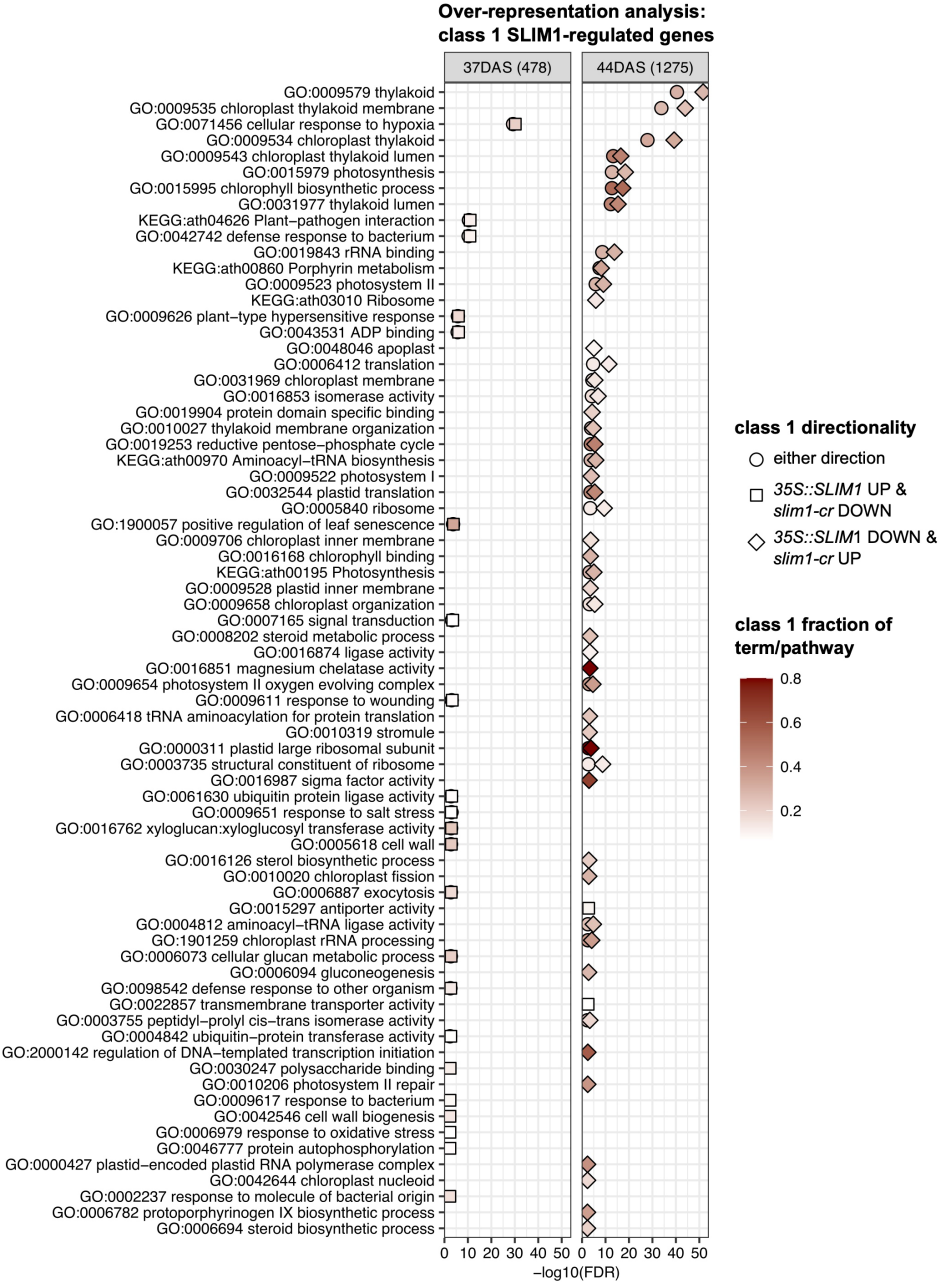
Over-representation analysis of GO terms and KEGG pathways. Over-representation analysis of GO terms and KEGG pathways annotated to class 1 genes was performed by Fisher’s exact test. In total, six sets of class 1 genes were tested: three directionality classes *35S::SLIM1* UP & *slim1-cr* DOWN (square), *35S::SLIM1* DOWN & *slim1-cr* UP (diamond), either directionality (circle) at two time points (37 DAS and 44 DAS). The gene universe was composed of the 20,899 genes with RNA-seq Wald test results in this study. GO terms and KEGG pathways with <5 or >500 genes in the gene universe were excluded from Fisher’s exact testing. All terms and pathways with Fisher’s exact FDR < 0.005 from at least one of the six class 1 gene sets are visualized. Fill color corresponds to the fraction of genes in a given GO term or KEGG pathway that are also class 1 SLIM1-regulated. The number of class 1 genes (either directionality) at each time point is shown in parentheses. Fisher’s exact text results for terms and pathways shown can be found in **Supplementary Table S3**.

In order to identify processes and functions that may be transcriptionally affected by SLIM1, we tested for over-representation of Gene Ontology (GO) terms and KEGG pathways in three sets of class 1 genes at 37 DAS and 44 DAS: class 1 genes that are positively regulated by SLIM1, class 1 genes that are negatively regulated by SLIM1, and all class 1 genes regardless of the response direction to SLIM1 (**Figure 6**). As expected, based on the expression of PAGs (**Figure 3C**), terms such as GO:0015979 photosynthesis, GO:0015995 chlorophyll biosynthetic process, GO:0016168 chlorophyll binding, GO:0009658 chloroplast organization, GO:0016851 magnesium chelatase activity, and GO:0006782 protoporphyrinogen IX biosynthetic process are over-represented in the set of *35S::SLIM1* DOWN & *slim1-cr* UP class 1 genes at DAS44 (**Figure 6**). These transcriptional changes may contribute to the lower chlorophyll content in *35S::SLIM1* at 51 DAS (**Figure 2A**). A highly over-represented GO term in our class 1 gene list is GO:0016851, magnesium chelatase (**Figure 6**). Magnesium chelatase inserts a Mg^2+^ into photoporphyrin IX, which is the first step of chlorophyll biosynthesis (Rissler et al., 2002). This GO term consists of five genes, and four of those genes are *35S::SLIM1* DOWN & *slim1-cr* UP class 1 genes at 44 DAS (**Supplementary Tables S2**, **S3**). Another GO term with a very high ratio of class 1 genes is GO:0000311, plastid large ribosomal subunit (**Figure 6**). Four of five genes annotated to this GO term are class 1 SLIM1 negatively regulated at 44 DAS (**Supplementary Tables S2**, **S3**). Among the GO terms that are over-represented in class 1 genes that are positively regulated by SLIM1 are GO:0005618 cell wall and GO:1900057 positive regulation of leaf senescence (**Figure 6**). This supports a role for *SLIM1* in regulating the timing of developmental senescence (**Figures 2**, **3**), and possibly the greater rosette area in *35S::SLIM1* (**Figure 1C**). Notably, no sulfur-related GO terms or KEGG pathways are over-represented in the list of class 1 SLIM1-regulated genes from analysis of plants grown on sufficient sulfur conditions (**Figure 6**).

### 3.5 *Cis*-regulatory sequences of the class 1 gene promoters

In this study, we identified 12 class 1 genes whose promoters were found by DNA affinity purification sequencing (DAP-seq) to be bound by SLIM1 protein (O’Malley et al., 2016) (**Table 2**). With Plant Regulomics (Ran et al., 2020), we searched for sequence motifs common to the promoters of those 12 genes (**Supplementary Table S4**). Using a *p* ≤ 0.05 cutoff, we identified the motif MNB1A (AAAGC), a C2H2 zinc finger binding site (**Table 2**). Additionally, the promoters of these 12 genes all contain SURE elements, a recently identified SLIM1 binding site (Rakpenthai et al., 2022), and 7 of the 12 have TEBS elements (**Table 2**). Searching for more class 1 genes whose promoters contain known SLIM1 binding motifs, SURE or TEBS, we identified seven additional genes (**Table 2**).

**Table 2:** *Cis*-regulatory element analysis of promoters of 19 selected class 1 SLIM1-regulated genes.

Gene ID	Gene name	DAP-seq^†^	SLIM1 EBM/M0680_1.02^‡^	SURE^§^	TEBS^¶^	MNB1A^††^
AT1G27100	*Actin cross-linking*	Y	Y	Y	Y	Y
AT1G30520	*AAE14*	Y	N	Y	N	Y
AT1G55260	*LTPG6*	Y	N	Y	Y	Y
AT2G34660	*ABCC2*	Y	Y	Y	Y	Y
AT3G08860	*PYD4*	Y	Y	Y	Y	Y
AT3G52840	*BGAL2*	Y	Y	Y	Y	Y
AT3G53310	*REM16*	Y	N	Y	Y	Y
AT3G58600	*NECAP-1*	Y	N	Y	N	Y
AT3G60140	*BGLU30*	Y	N	Y	N	Y
AT4G01000	*SDE2*	Y	N	Y	N	Y
AT4G11670	*DUF810*	Y	Y	Y	Y	Y
AT5G46830	*BHLH28/MYC5*	Y	N	Y	N	Y
AT3G56040	*UGP3*	N	N	Y	N	Y
AT4G24770	*RBP31*	N	N	Y	N	Y
AT4G31500	*CYP83B1*	N	N	Y	Y	Y
AT5G24090	*CHIA*	N	N	Y	N	Y
AT5G25820	*Exostosin*	N	N	Y	Y	Y
AT5G38940	*RMLC-like*	N	N	Y	N	Y
AT5G43780	*APS4*	N	N	Y	N	Y

Promoter region defined as between the transcription start site and 1,000 bp upstream. Genes whose promoter was bound by SLIM1 protein by DNA affinity purification sequencing (DAP-seq) are indicated with Y for yes, all others with N for no. Presence of binding site, cis-regulatory element, or motif in the promoter is indicated with Y for yes or N for no.

† O’Malley et al. (2016).

‡ SLIM1 enriched binding motif/ M0680_1.02 identified by DAP-seq (O’Malley et al., 2016) [A/C/G/T]AATG[T/A]A[C/T]CT[A/C/G/T].

§ SURE *cis*-regulatory element GAGAC consensus sequence.

^¶^TEBS *cis*-regulatory element A[T/C]G[A/T]A[C/T]CT consensus sequence.

†† MNB1A motif AAAGC consensus sequence.

These 19 genes have the strongest evidence of being directly regulated by SLIM1 in our experimental system. Here, we briefly describe the proteins encoded by the genes in **Table 2**. Two of the 19 genes code for proteins associated with anthocyanin metabolism. ABCC2 (AT2G34660) is a transporter of acetylated anthocyanins across the vacuolar membrane (Behrens et al., 2019), and SDE2 (AT4G01000) is a ubiquitin-like superfamily protein that has been shown to indirectly regulate anthocyanin biosynthesis (Xie et al., 2017). Two genes encode proteins involved in electron transport in photosystem I. AAE14 (AT1G30520) is an enzyme in the biosynthetic pathway for phylloquinone, an essential photosystem I electron carrier (Kim et al., 2008). RBP31 (AT4G24770) is an RNA-binding protein involved in chloroplastic RNA editing (Okuzaki et al., 2021), especially RNAs encoding subunits of the NDH complex, which mediates photosystem I cyclic electron transport (Lenzen et al., 2020). Four genes encoding sulfur metabolism-related proteins are in the set of 19. These are *APS4* (AT5G43780), *UGP3* (AT3G56040), *CYP83B1* (AT4G31500), and *BGLU30* (AT3G60140). APS4 is a sulfate adenyltransferase that activates sulfate for reduction to sulfide or for sulfonation (Hatzfeld et al., 2000). UGP3 is a UDP-glucose pyrophosphorylase and is required for synthesis of sulfoquinovosyldiacylglycerol lipids, which are components of chloroplast membranes (Okazaki et al., 2009). CYP83B1 catalyzes an intermediate step in the biosynthesis of indolic glucosinolates (Goto et al., 2023), and BGLU30 is a beta-glucosidase involved in glucosinolate catabolism. *BGAL2* (AT3G52840) encodes an apoplast-localized beta-galactosidase. Although BGAL2 functions are not well characterized, expression of other BGAL family proteins are associated with cell wall expansion and cell wall remodeling in a variety of tissues (Chandrasekar and Van Der Hoorn, 2016). Genes known to be involved in abiotic and biotic stress responses were also identified. These include *PYD4* (AT3G08860), *LTPG6* (AT1G55260), and *CHIA* (AT5G24090). *PYD4* encodes a beta-alanine aminotransferase that performs the final enzymatic step in the synthesis of beta-alanine from propanoate (Parthasarathy et al., 2019). Beta-alanine accumulates in plants exposed to many biotic and abiotic stresses. LTPG6 is an apoplast-localized, non-specific lipid transport protein that has been shown to affect penetration of the epidermal cell wall by some fungi (Fahlberg et al., 2019). CHIA catalyzes bacterial cell wall peptidoglycan breakdown in order to trigger plant immunity responses (Liu et al., 2014). The list of genes in **Table 2** also includes two transcription factors: *REM16* (AT3G53310) and *bHLH28/MYC5* (AT5G46830), which are involved in regulation of photoperiodism and flowering (GO:2000028) and diverse jasmonate-mediated processes (Song et al., 2017), respectively. Little is known functionally about several of these 19 putative SLIM1-target genes beyond what we can infer from their protein family membership. RMLC-like (AT5G38940) is a member of the functionally diverse protein superfamily, Cupins (Dunwell, 1998), which includes seed storage proteins and germins. AT5G25820 encodes an exostosin family protein and is thereby predicted to have glycosyltransferase activity (GO:0016757). Functional annotations for *NECAP-1* (AT3G58600) include clathrin vesicle coat (GO:0030125) and vesicle-mediated transport (GO:0016192) and are inferred based on phylogeny. Among these genes in **Table 2**, two code for proteins with domains of unknown function (DUF). AT1G27100 protein contains two DUF569 domains and AT4G11670 protein contains a DUF810 domain.

We performed promoter analysis of class 1 genes to explore whether particular GO terms or KEGG pathways are in general being regulated directly or indirectly by SLIM1. Initially, we performed promoter analysis for the genes annotated to GO terms with a minimum 0.66 fraction of class 1 genes (**Figure 6**), which were GO:0016851 magnesium chelatase activity (four of five genes are class 1) and GO:0000311 plastid large ribosomal subunit (four of six genes are class 1). None of the eight genes that are annotated to these terms contain SURE and TEBS elements, or SLIM1 binding sites in their promoters. None of the eight genes annotated to those GO terms were identified by DAP-seq to be bound by SLIM1 (O’Malley et al., 2016). This indicates that SLIM1 may have an indirect regulatory effect on the genes of these GO terms. At 37 DAS, terms over-represented in class 1 genes that are not linked to the observed phenotypes such as senescence, development, and growth include KEGG:ath04626 plant–pathogen interaction, GO:0009611 response to wounding, GO:0009651 response to salt stress, GO:0098542 defense response to other organism, GO:0009617 response to bacterium, and GO:0006979 response to oxidative stress. Promoter analysis of the 149 genes annotated to the six GO terms mentioned above revealed that one promoter has the SLIM1 enriched binding motif (O’Malley et al., 2016) and two promoters contain SURE elements [*DIN2* (*AT3G60140*) and *CHIA* (*AT5G24090*)]. Since 35S::SLIM1 lines develop and senescence faster, we performed promoter analysis for the genes belonging to GO:1900057 positive regulation of leaf senescence. None of the 18 genes annotated to this term contain known SLIM1 binding sites in their promoters. Taken together, the promoter analysis suggests that most of the genes in the selected over-represented GO terms and KEGG pathways are unlikely to be regulated directly by SLIM1 binding to their promoters.

## 4 Discussion

SLIM1 is an ethylene insensitive 3-like (EIL) family TF that plays an important role in the transcriptional response to −S (Maruyama-Nakashita et al., 2006; Wawrzyńska and Sirko, 2014; Rakpenthai et al., 2022; Apodiakou and Hoefgen, 2023), to arsenic (Jobe et al., 2021), and to cadmium (Yamaguchi et al., 2020) in *A. thaliana*. However, *SLIM1* has not been well studied in the context of sufficient nutrient/unstressed conditions. We grew *A. thaliana* Col-0, *EV, slim1-cr*, and multiple T2 generation *35S::SLIM1* lines in soil under long-day conditions and monitored their growth for 7 weeks (to 51 DAS). We found that the plants overexpressing *SLIM1* have 1.5- to 3-fold greater rosette area across a wide developmental window (**Figure 1C**). Additionally, late in development *35S::SLIM1* leaves have chlorotic tips, a sign of senescence onset (Watanabe et al., 2013), have darker and purplish pigmentation, and are more curled compared to the control lines (**Figure 2A**). Pigments such as anthocyanins and chlorophylls were found to be altered in *35S::SLIM1* rosettes compared to the controls at this late time point (**Figures 2B**, **3D**). Degradation of chlorophyll (Hörtensteiner, 2006; Gao et al., 2016), decline of photosynthesis (Masclaux-Daubresse et al., 2010; Lichtenthaler, 2012; Tamary et al., 2019), and accumulation of anthocyanins (Hoch et al., 2001; Diaz et al., 2006; Liu et al., 2018) are hallmarks of the early stages of leaf senescence, suggesting that *35S::SLIM1* lines enter senescence earlier than controls. It has been shown via yeast 1 hybrid that SLIM1 binds to the promoter of *PAP1/MYB75* and as a result promotes anthocyanin accumulation under –S, while anthocyanin content in *slim1-cr* is similar under S-deficient and S-sufficient conditions (Wawrzyńska et al., 2022). These data, in combination with the transcripts of *PAP1/MYB75* in *35S::SLIM1* (**Figure 2D**) and the anthocyanin and flavonoid accumulation in *35S::SLIM1* (**Figures 2B, C**), suggest that while SLIM1 may not be necessary for normal anthocyanin accumulation during senescence or S-deficient conditions, higher levels of SLIM1 promote anthocyanin production by directly regulating *PAP1/MYB75*. Moreover, the earlier bolting in *35S::SLIM1* is an indication that *35S::SLIM1* plants transition earlier into the reproductive phase, demonstrating accelerated development. The transition from the vegetative to the reproductive phase is tightly linked to senescence (Balazadeh et al., 2008; Hinckley and Brusslan, 2020).

*35S::SLIM1* plants have greatly increased rosette area throughout the vegetative growth phase (**Figures 1B, C**). Epidermis images revealed that the size (surface area) of epithelial cells was unaltered in *35S::SLIM1* genotypes compared to controls (**Supplementary Figures S2D, E**), indicating that the larger rosette area in *35S::SLIM1* plants is not due to the increased epithelial cell size. Leaf growth is affected by a sequence of linked processes, such as cell proliferation, meristemoid division, and cell expansion (Gonzalez et al., 2012). Differential regulation of cell proliferation during leaf growth can affect final leaf size. Genes involved in cell proliferation were analyzed for differential expression in our RNA-seq dataset (**Supplementary Figure S2F**, **Supplementary Table S1**). While a substantial number of cell proliferation genes were found to be differentially expressed in *35S::SLIM1*, overall, the differences relative to Col-0 were concentrated at the later time points (37 and 44 DAS), and therefore cannot explain the consistently larger leaves in *35S::SLIM1* lines from at least 16 DAS (**Figure 1C**). In Arabidopsis, the extent of cell division of the leaf primordium founder cells can also affect the final leaf size through differences in cell number (Vercruysse et al., 2021). It may be that an important cause of larger rosettes in *35S::SLIM1* plants can be traced back to differences in these primordium founder cells.

SLIM1 is a key regulator of sulfur deficiency responses, and sulfur metabolism directly impacts redox regulation, which may contribute to the plant development and leaf area phenotypes observed in the *35S::SLIM1* lines (**Figures 1**–**3**). Therefore, we assessed whether *35S::SLIM1* plants have altered sulfur metabolism in the sufficient sulfur conditions used in our study. Over-representation analysis of class 1 SLIM1-regulated genes did not identify any GO terms or KEGG pathways related to sulfur metabolism (**Figure 6**), suggesting that sulfur metabolism is not broadly regulated on the transcriptional level by SLIM1 under our sulfur-sufficient experimental conditions. This is similar to the recent finding that the majority of genes involved in sulfur metabolism/assimilation are not differentially expressed in *eil3/slim1* mutant under sulfur-sufficient conditions (Dietzen et al., 2020). Moreover, the sulfate content in *35S::SLIM1* plants was very similar to Col-0 and *EV* at all time points tested (**Supplementary Figure 4A**). Cysteine levels tended to be higher in *35S::SLIM1* lines than Col-0, but were only consistently statistically different at 30 DAS (**Supplementary Figure 4B**). Glutathione is an important S-containing redox buffering compound (Joshi et al., 2019), and it is thought to play an important role in regulating plant development (Rouhier et al., 2015). Like cystine, glutathione tended to be higher in *35S::SLIM1* plants but is only consistently and clearly accumulated at 30 DAS. However, at this time point, glutathione was elevated in both *slim1-cr* and *35S::SLIM1* plants (**Supplementary Figure 4B**), so it is unlikely to be a major contributor to the early bolting phenotype observed in the *35S::SLIM1* lines (**Figure 3A**) or directly related to the consistently greater size of *35S::SLIM1* plants across our 4-week time course (**Figure 1B**). Another major pool of organic sulfur in Arabidopsis is glucosinolates. *35S::SLIM1* lines did not exhibit consistently altered glucosinolate levels relative to controls (**Supplementary Figure 4D**), and at 30 DAS and 37 DAS, glucosinolate levels were similar in *slim1-cr* and *35S::SLIM1* lines. Transcripts of the majority of sulfate assimilation pathway, sulfate transporters, and OAS-cluster genes are regulated similarly in *slim1-cr* and *35S::SLIM1* lines (**Supplementary Figure 4E**). Taken together, these data suggest that overexpression of SLIM1 TF in our sulfur-sufficient growth conditions does not strongly affect sulfur metabolism.

SLIM1 has been shown to regulate genes involved in ROS/redox signaling functional categories under S-sufficient conditions (Dietzen et al., 2020), and redox regulation plays a role in plant growth and developmental transitions. Therefore, we analyzed the expression pattern of 56 genes annotated as (cellular) response to ROS (GO:0000302 and GO:0034614), response to redox state (GO:0051775), and antioxidant activity (GO:0016209) at the three time points in our RNA-seq experiment (**Supplementary Figure 4F**). At 30 DAS, when approximately 60% of *35S::SLIM1* plants but less than 25% of *slim1-cr* and control plants have visibly bolted (**Figure 3A**), only one gene in the ROS/redox related set, *COR78* (AT5G52310), appears to be regulated by SLIM1. By 37 DAS, *35S::SLIM1* plants show some transcriptional signs of having entered developmental senescence (**Figure 3B**), and five genes (AT4G17490, AT3G01420, AT1G19020, AT4G02380, and AT4G18880) show differential expression patterns consistent with positive regulation by SLIM1. Among these five, *SDA1* (AT1G19020) was also found to be differentially expressed under sulfur-sufficient conditions in *eil3* (Dietzen et al., 2020). Several ERF transcription factors have been previously shown to be differentially regulated in *eil3* under S-sufficient conditions, but not the specific ERF found in our RNA-seq experiment, *ATERF6*. By 44 DAS, *35S::SLIM1* plants show some visible signs of senescence and many expression changes in PAGs and SAGs (**Figures 3B, C**). Among the ROS/redox-related genes, more show clear expression patterns consistent with SLIM1 regulation at 44 DAS than at earlier time points. This may be due to the strong link between ROS and plant aging/senescence (Pole et al., 2016).

Given the purple/pink pigmented phenotype (**Figure 2A**) and earlier bolting time (**Figure 3A**) in *35S::SLIM1* lines, we suspected that *35S::SLIM1* plants may develop faster and enter developmental senescence earlier. The first physiological processes affected during senescence are chloroplast function (Masclaux, 2000) and photosynthesis (Guo et al., 2004; Gregersen et al., 2013; Thomas, 2013). PAGs (Hye et al., 2004; Hörtensteiner, 2006; Gao et al., 2012; Motohashi et al., 2012; Thomas, 2013; Liang et al., 2019), known to be downregulated during leaf senescence, are downregulated in *35S::SLIM1* compared to Col-0 (**Figure 3C**), and to a lesser extent induced in *slim1-cr*. PAGs downregulation is consistent with the decline in the total chlorophyll content between 37 DAS and 44 DAS (**Figure 3D**). GO terms related to photosynthesis, chlorophyll, and chloroplast are overrepresented in class 1 genes negatively regulated by SLIM1 (**Figure 6**) at 44 DAS. Expression of PAGs is known to be negatively correlated with expression of SAGs (Buchanan-Wollaston et al., 2005; Lichtenthaler, 2012). SAGs (Hye et al., 2004; Li et al., 2012; Wu et al., 2012; Sakuraba et al., 2014; Hsu et al., 2022) and/or autophagy genes (Sakuraba et al., 2014) are known to accumulate during leaf senescence. SAGs are induced in *35S::SLIM1* (**Figure 3B**), indicating that *35S::SLIM1* has entered developmental senescence earlier than the other genotypes.

The characteristic phenotype with the purple/pink pigmentation observed in *35S::SLIM1* plants (**Figure 2A**) directed our interest to identify genes that are most likely regulated directly by SLIM1 (**Table 2**). *ABCC2* (*AT2G34660*) is a class 1 gene at 44 DAS, which is positively regulated by SLIM1 and is a SLIM1&DAP-seq target (O’Malley et al., 2016). ABCC2 is an ABC transporter, specifically an anthocyanin transporter, and is involved in the vacuolar transport of acylated anthocyanins at the vegetative stage (Behrens et al., 2019; Dean et al., 2022). Anthocyanins, after they are synthesized in the cytoplasm, are modified (methylated, glycosylated, or acylated), and are sequestered in vacuoles where the environment is acidic. As a result, oxidation of the anthocyanins is prevented and they are stabilized to function as pigments (Dean et al., 2022). *ABCC2* induction in *35S::SLIM1* suggests increased flux of anthocyanins from the cytoplasm into the vacuole where they are stabilized. Furthermore, *EIN3* transcripts have been shown to accumulate during leaf senescence and constitutive overexpression of *EIN3* accelerates leaf senescence, while leaf senescence is delayed in *ein3* mutants (Li et al., 2013; Kim et al., 2014). EIN3 positively regulates leaf senescence through *NAC6* and *NAP* activation (Kim et al., 2014). *NAC6* and *NAP* were also upregulated in the *35S::SLIM1* lines, but *EIN3* is not induced relative to Col-0 (**Figure 3B**). Therefore, the early senescence phenotype in *35S::SLIM1* is not mediated by *EIN3* induction. EIN3 can form a heterodimer with SLIM1, which inhibits SLIM1 binding to DNA (Wawrzyńska and Sirko, 2016) but the levels of SLIM1 protein in the *35S::SLIM1* lines may be high enough that sufficient SLIM1 remains unbound to EIN3 and is thereby free to bind gene promoters. Therefore, SLIM1 can now positively regulate, directly or indirectly, *NAC6* and *NAP1*.

NO_3_^−^ content in *35S::SLIM1* is lower than in the controls in the early time points, and it decreases more than the controls do as the plants age (**Figure 4A**). Transporters such as *NRT1;5*, a long-distance root-to-shoot transporter (Chen et al., 2021), and *NRT1;7*, a source-to-sink transporter (Fan et al., 2009) and a class 1 SLIM1-regulated gene, are upregulated in *35S::SLIM1* at 44 DAS. Taken together, these data are preliminary indications that NO_3_^−^ may be undergoing remobilization to the sink organs earlier than in Col-0 due to senescence. Additionally, *NRT2;5*, which is induced under N-starvation conditions, is 26-fold upregulated in *35S::SLIM1* (**Figure 4B**) at a time point when NO_3_^−^ content in the *35S::SLIM1* rosette is extremely low (**Figure 4A**). Also at 44DAS, six of the nine *NLP* TFs in Arabidopsis, which are considered key TFs in NO_3_^−^ sensing and the NO_3_^−^ signaling pathway (Krapp, 2015; Mu and Luo, 2019), are induced in both *35S::SLIM1* lines compared to the control lines (**Figure 4B**). *NLPs* are induced when the plants grow under chronic NO_3_^−^ starvation (Krapp, 2015; Luo et al., 2021). The *NLP* expression data (**Figure 4B**) are consistent with the low NO_3_^−^ content detected in *35S::SLIM1* (**Figure 4A**).

In addition to genome-wide *in vitro* SLIM1 binding data (DAP-seq), multiple studies have identified motif sequences that are involved in SLIM1-mediated gene regulation under −S (Wawrzyńska and Sirko, 2016; Rakpenthai et al., 2022), including the SURE, TEBS, and UPE-box. Recently, it has been shown that SURE *cis*-regulatory element(s) are important for SLIM1 binding to the promoters of *SDIs in vitro* (Rakpenthai et al., 2022) and are important for induction of –S responsive genes that repress glucosinolate biosynthesis in Arabidopsis (Aarabi et al., 2016). Notably, all 12 of the genes with class 1 differential expression behavior here in unstressed conditions that were also identified as SLIM1 binding targets by DAP-seq have SURE element(s) in their promoter. In total, 19 class 1 genes were found to contain SURE element(s) and/or TEBS in their promoter sequences (**Table 2**). Taken together, these data indicate that SLIM1 binding to a gene promoter is unlikely to depend on a single particular *cis*-regulatory element or motif. Furthermore, SLIM1 binding to promoters might also depend on PPI with other trans-acting transcriptional regulators and with chromatin at potential target genes. PPI and the post-translational modifications that influence them are often tissue-, development-, and condition-dependent. For instance, SLIM1 DNA binding is inhibited by heterodimer formation with EIN3 (Wawrzyńska and Sirko, 2016). In Col-0, *EIN3* is induced during senescence (Li et al., 2013; Kim et al., 2014), while *SLIM1* expression remains stable (Van Der Graaff et al., 2006). Perhaps EIN3:SLIM1 complex formation reduces SLIM1 binding to PAG promoters, thereby slowing down senescence. Twenty PAGs (**Figure 3C**) are class 1 genes and five PAGs*, LHCA2, LHCA3, LHCA4, SIGF*, and *PTAC14*, contain SLIM1 enriched binding motifs (from DAP-seq). For the complete motif analysis from Plant Regulomics, please see **Supplementary Table S4**.

## 5 Conclusions

In this study, we show that overexpression of *SLIM1* in Arabidopsis results in plants with larger rosettes and earlier maturity. Shorter time to maturity is an important trait to farmers because it reduces exposure to various environmental stresses, such as heat and drought at the end of the growing season. Moreover, faster crop maturation can allow farmers to plant additional crops in the same field in the same growing season. Increased leaf area may benefit farmers as well by increasing photosynthetic efficiency and suppressing weeds without the use of additional herbicides. We look forward to future studies that will test whether overexpression of *SLIM1* paralogs in crop species results in the same beneficial phenotypes that we observe here in Arabidopsis.

It is not possible with the available data to determine precisely how many of the 1,731 class 1 genes identified in this study are directly regulated by SLIM1 via promoter binding *in vivo*. However, the fact that only 7 class 1 genes contain SURE elements in their promoter and 307 contain the DAP-seq SLIM1 enriched binding motif suggests that the expression of most class 1 genes is regulated by SLIM1 indirectly in our system. Direct modulation of expression by overexpressed SLIM1 triggers further gene expression responses in a pleiotropic manner; it eventually affects many genes and hence steers a variety of physiological responses. Post-translational modifications and PPI are likely important for *SLIM1* function. For future research, attention should be paid to identify new SLIM1 protein interaction partners and post-translational modifications on SLIM1 protein that contribute to the growing list of gene regulatory functions of SLIM1.

## Data availability statement

The RNA-seq data discussed in this publication have been deposited in NCBI’s Gene Expression Omnibus (Edgar et al., 2002) and are accessible through GEO Series accession number GSE243846 (https://www.ncbi.nlm.nih.gov/geo/query/acc.cgi?acc=GSE243846). These data include the clean read FASTQ files for each sample, the raw read count per gene table (**Supplementary Data Sheet S1**), and the normalized read count per gene table (**Supplementary Data Sheet S2**).

## Author contributions

AA: Formal analysis, Investigation, Project administration, Validation, Writing – original draft, Writing – review & editing. SA: Investigation, Writing – review & editing. RH: Conceptualization, Funding acquisition, Project administration, Supervision, Writing – review & editing. SW: Data curation, Formal analysis, Supervision, Visualization, Writing – review & editing.
