# Supplementary material for: Overexpression of SLIM1 transcription factor accelerates vegetative development in Arabidopsis thaliana
Source: Front Plant Sci. 2024 Mar 20;15:1327152. doi: 10.3389/fpls.2024.1327152 (PMC10988502; doi:10.3389/fpls.2024.1327152)
Supplement: Supplementary file 4 [file Image_1.pdf]

Methods S1: primers and qPCR methods

primer sequences

primer name	primer sequence (5' to 3')
Attb1 - SLIM1	GGGGACAAGTTTGTACAAAAAAGCAGGCTTCATGGGCGATCTTGCTATGTCC
Attb2 – SLIM1	GGGGACCACTTTGTACAAGAAAGCTGGGTCCTAAGCTCCAAACCATGAGAAATC
prom35S F	ACGCACAATCCCACTATCCTTC
SLIM1 R1	CTAAGCTCCAAACCATGAGAAATC
SLIM1 R2	CTGAGGCAACTCACTCCCTG
SLIM1 qPCR F *	ATCCGTTGGAGAAAGGGACG
SLIM1 qPCR R *	GGCTTTTAGGCAGACCGAGT
UBQ10 qPCR F	GGCCTTGTATAATCCCTGATGAATAAG
UBQ10 qPCR R	AAAGAGATAACAGGAACGGAAACATAGT
Hyg qPCR F	GTGCTTTCAGCTTCGATGTAG
Hyg qPCR R	GAAGAACAGCGGGCAGTTCGG
Hyg probe	GTGCTTGACATTGGGGAGTTCAG
GI qPCR F	TTCTTCTGCGGGCAACTGAT
GI qPCR R	TCGACCACTGCTAGTCCAGA
GI probe	GAGCTACTTGAAGCCACGGCAAGAG

* Primers annealing on the SLIM1 deleted 5 prime end CDS (F) and on the CDS next to the deletion 3 prime end. This set of primers was used in order to produce the Figure 1a.

qRT-PCR reaction preparation

component	volume
cDNA sample (1:9)	0.5 µl
SYBR Green PCR Master Mix	2.5 µl
Primer mix (F + R) 1µM	2 µl

PCR program for qRT-PCR

Initialization (95°C) was followed by denaturation cycles (95°C) and primer annealing (60°C).

**RT-PCR cycle**
50 ºC 2'
95 ºC 10'
95 ºC 15'' **X 40** 60 ºC 30''
95 ºC 15''
60 ºC 15'' -> 95 ºC 15'' (**Dissociation stage)**

TaqMan reaction preparation

component	volume
2x TaqMan Universal PCR Master Mix	5 µl
Hyg qPCR F (10µM)	0.5 µl
Hyg qPCR R (10µM)	0.5 µl

Hyg probe (2µM)	0.5 µl
GI qPCR F (10µM)	0.5 µl
GI qPCR R (10µM)	0.5 µl
GI probe (2µM)	0.5 µl
DNA sample (100 ng/µl)	0.5 µl
ddH2O	1.5 µl

PCR program for TaqMan

**RT-PCR cycle**
50 ºC 2’
95 ºC 2’
95 ºC 15’’<br>**X 40**<br>60 ºC 30’’
95 ºC 15’’
60 ºC 30’’ -> 95 ºC 15’’ (**Dissociation stage)**
