# Supplementary material for: Overexpression of SLIM1 transcription factor accelerates vegetative development in Arabidopsis thaliana
Source: Front Plant Sci. 2024 Mar 20;15:1327152. doi: 10.3389/fpls.2024.1327152 (PMC10988502; doi:10.3389/fpls.2024.1327152)

Supplementary Figure 1: photo of rosettes at 37 DAS and 44 DAS

Photo of representative rosettes at 37 DAS (A) and 44 DAS (B).

A

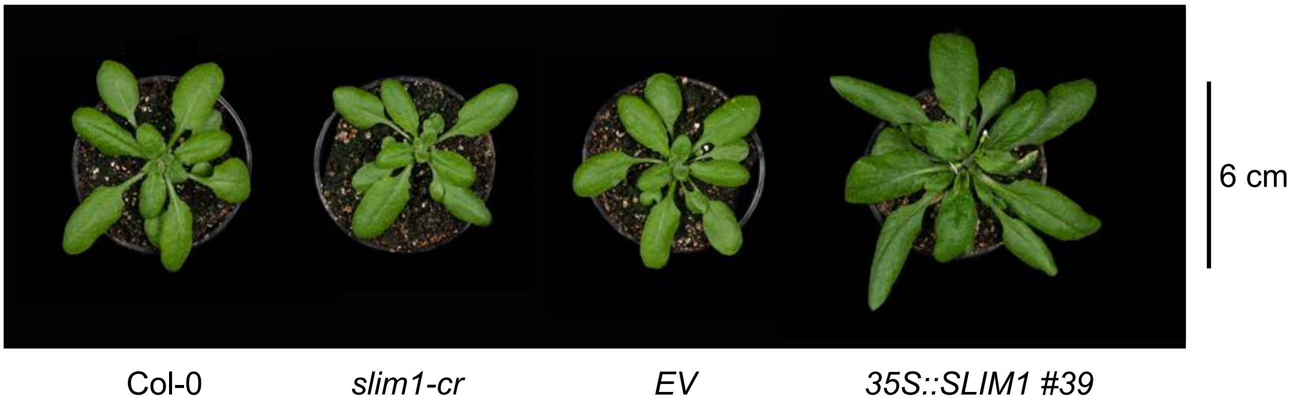

B

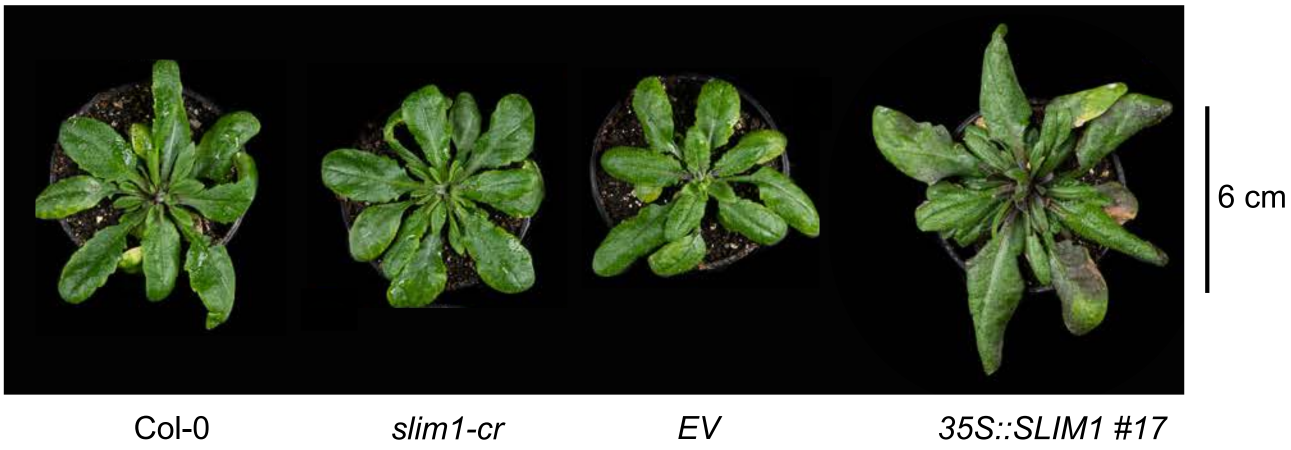

Supplementary Figure 2: rosette biomass, leaf microscopy, and expression of cell proliferation genes
A) Rosette fresh weight (FW) biomass relative to Col-0 at each timepoint. Points represent the mean of 5 rosettes, and error bars show the SEM. T-test results can be found in Table S1. (B) Histological cross-sections of mature leaves at 35 DAS. Samples were stained with 0.05% of Toluidine blue in water. Scale = 50 µm. (C) Number of cells (N) normalized to cross-section area (mm^2). Bar height corresponds to mean of 6-9 leaves, and error bars represent the standard error of the mean (SEM). (D) Comparison of leaf epithelial cells of mature leaves at 30 and 37 DAS. Scale = 37 µm. (E) The distributions of epithelial cell area for 40 cells per genotype are depicted by boxplot. The boxes contain cell areas between the 25th and 75th percentiles, whiskers correspond to the 10th and 90th percentile. (C, E) Statistical significance was assessed using t-tests, and p-values were adjusted by Benjamini-Yekutieli method. Compact Letter Display (CLD) identifies lines that are statistically different (adjusted $p \leq 0.05$) from each other. (F) Heatmap representation of gene expression differences determined by RNA-seq. Genes shown are annotated to various cell proliferation genes (Vercruysse et al., 2021). Heatmap color corresponds to log2 of the fold change (log2FC) relative to Col-0 at each timepoint determined from 3 biological replicates. Wald test results for genes shown can be found in Table S1.

A

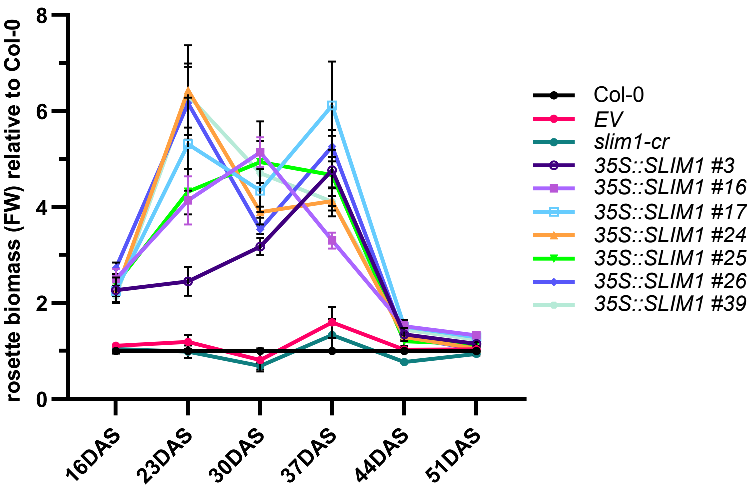

B

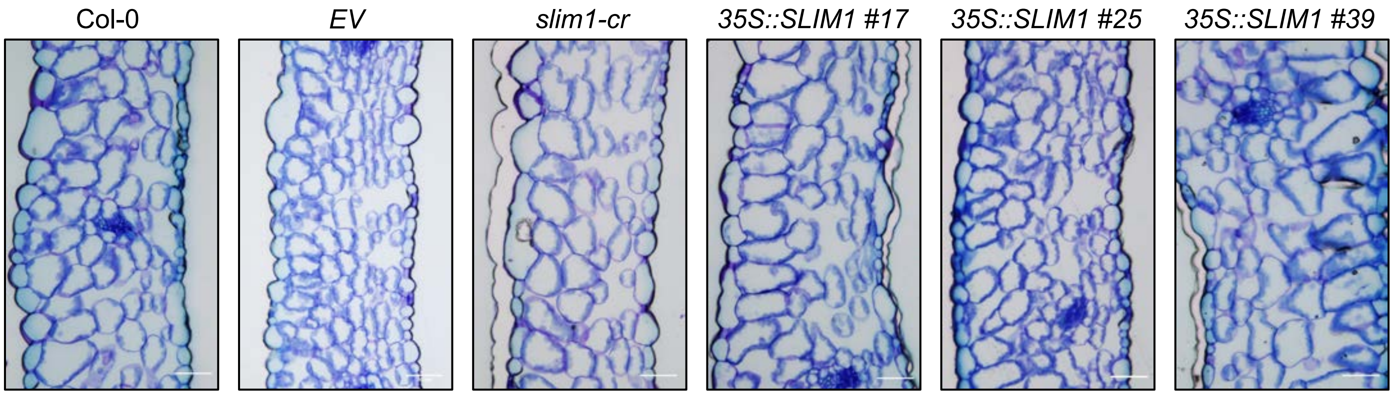

Supplementary Figure 2

C

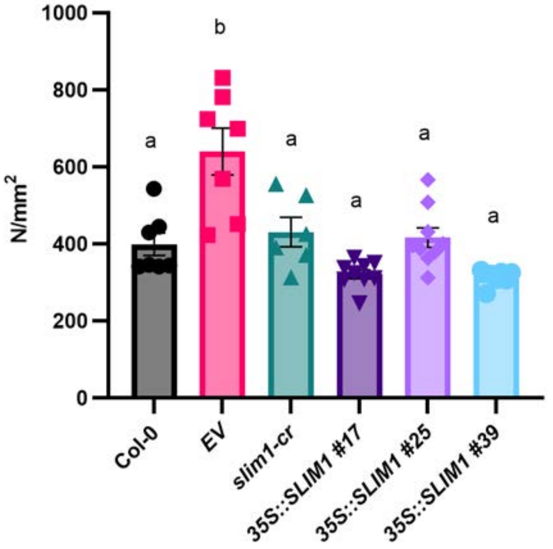

D

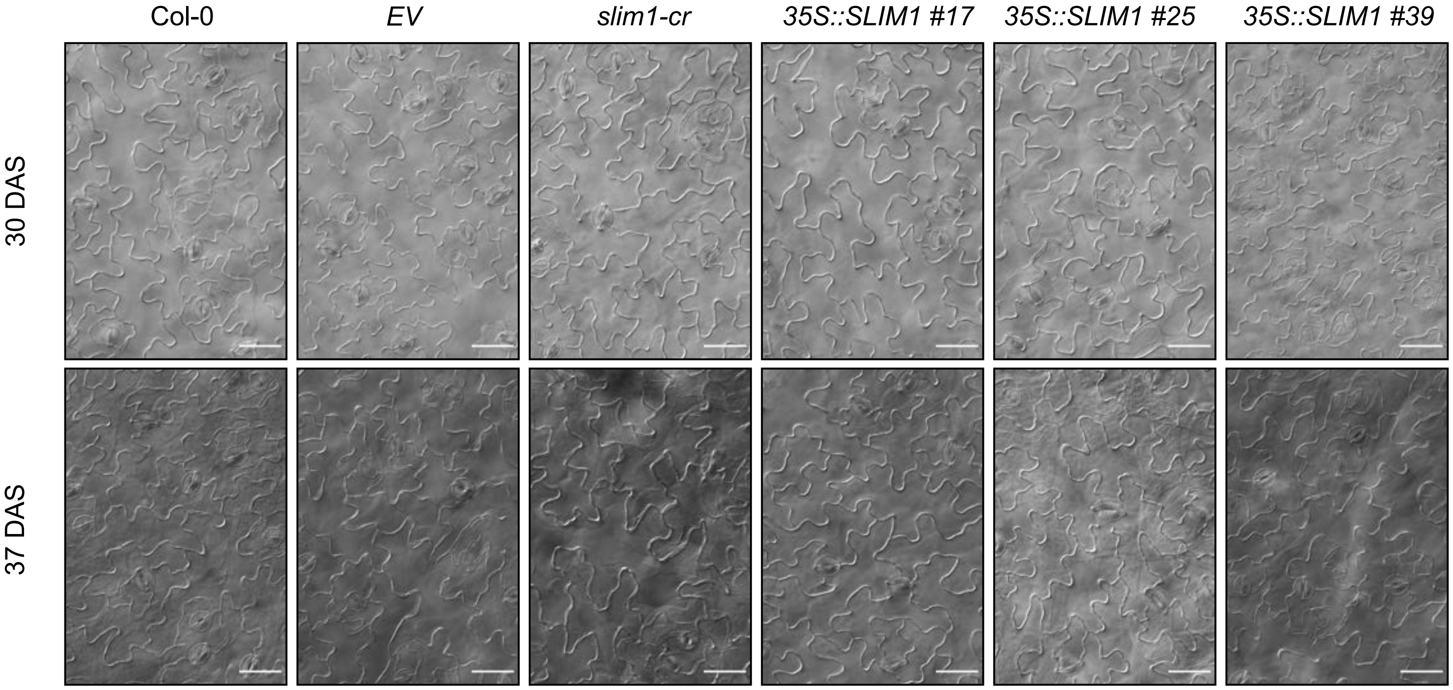

E

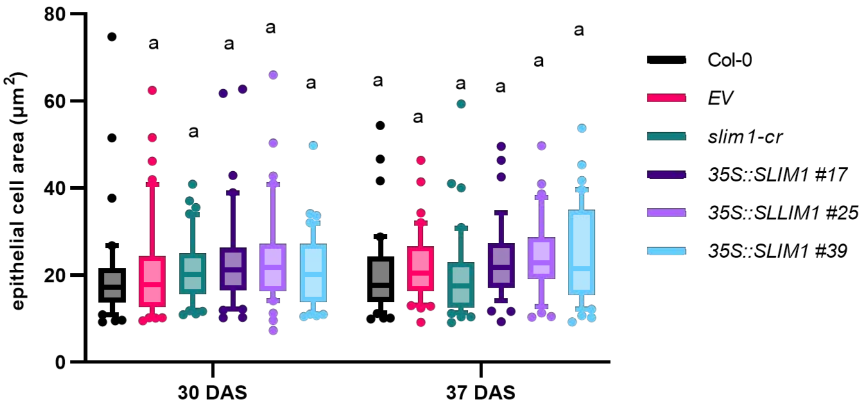

Supplementary Figure 2

F

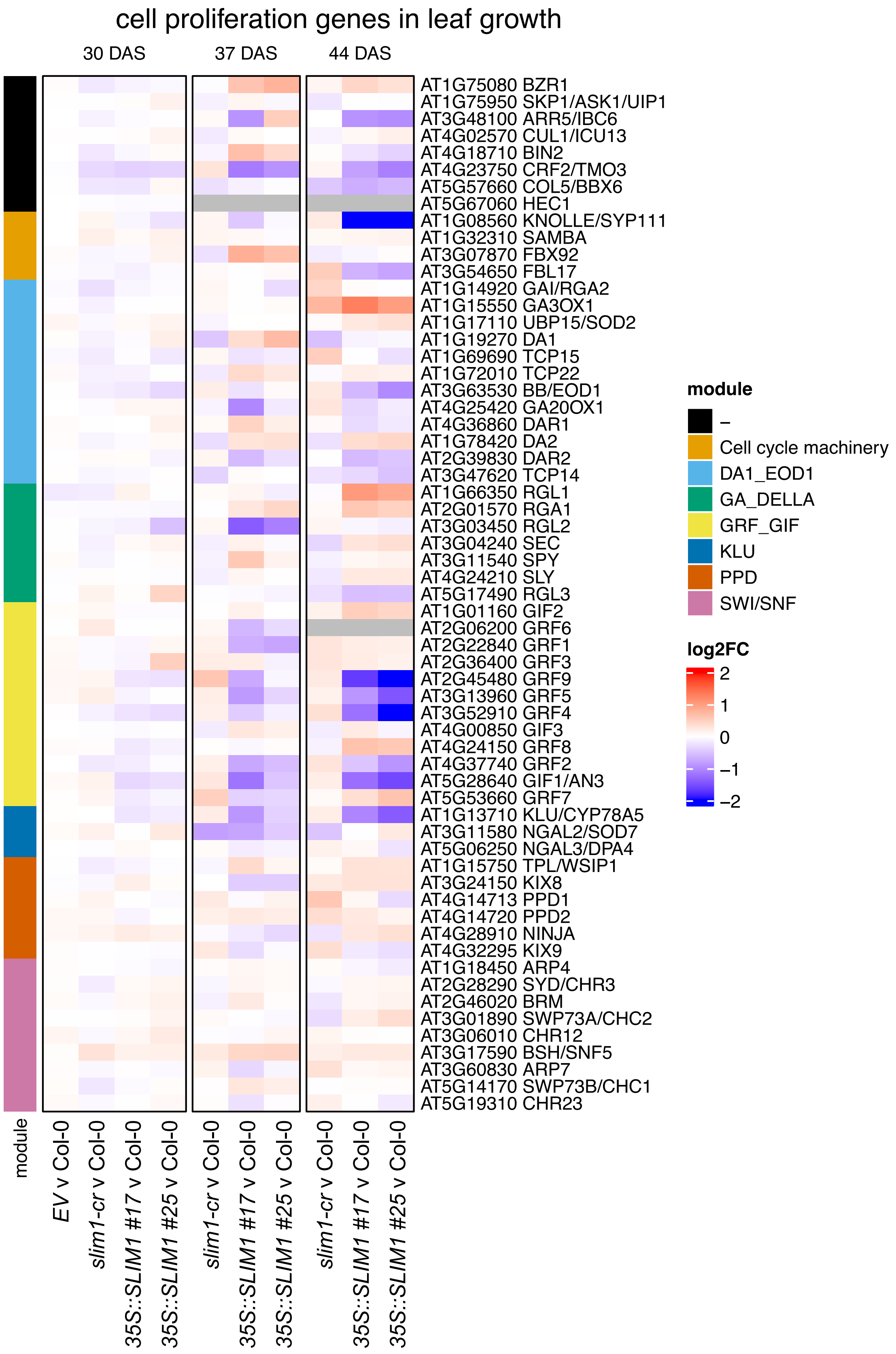

Supplementary Figure 3: Differentially expressed gene count

The number of differentially expressed genes (DEG) at each timepoint in each contrast is shown. All DEG have an adjusted p-value < 0.05. The total number of genes tested for differential expression by Wald test at each timepoint is indicated in parentheses.

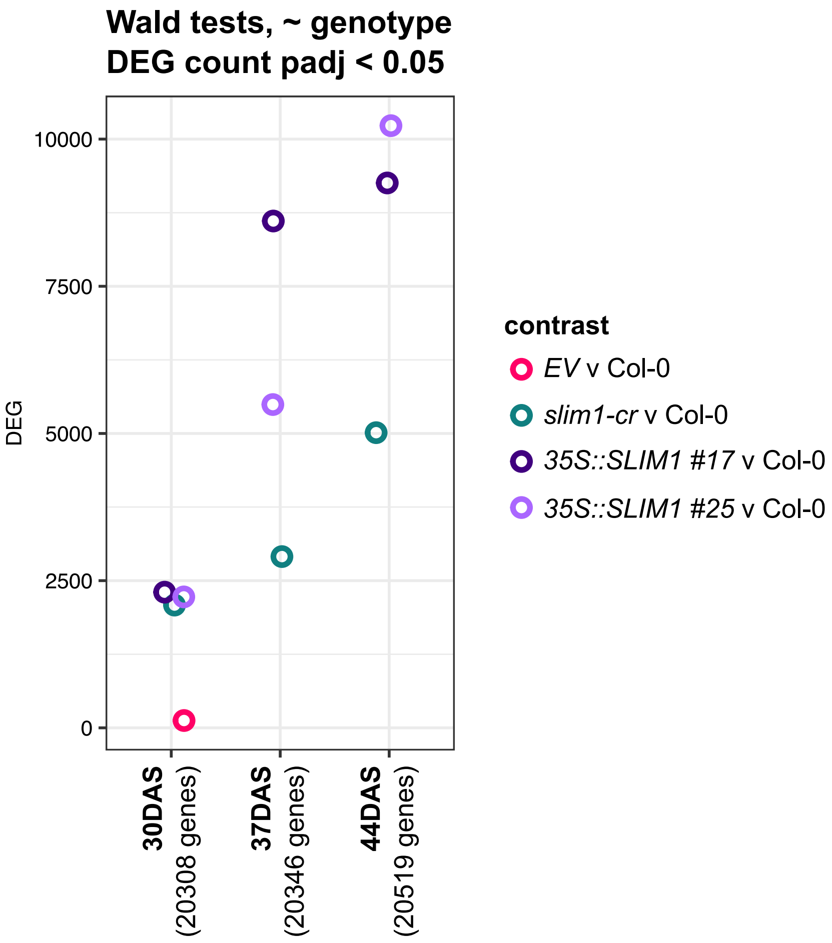

Supplementary Figure 4: sulfur metabolism and ROS/redox related gene expression

The concentration of major sulfur-containing compounds (A-D) in rosette tissue normalized to FW is shown. Bar height corresponds to mean of 3 biological replicates, dots correspond to the concentration determined in individual samples, and error bars represent the standard error of the mean (SEM). Statistical significance was assessed using t-tests within each timepoint. T-test results can be found in Table S1. P-values were adjusted by Benjamini-Yekutieli method. Compact Letter Display (CLD) identifies lines that are statistically different (adjusted $p \leq 0.05$) from each other at each timepoint. Heatmap representation of expression differences determined by RNA-seq of sulfur metabolism related genes (E) and genes annotated to (cellular) response to ROS (GO:0000302, GO:0034614), response to redox state (GO:0051775), and antioxidant activity (GO:0016209) (F). Heatmap color corresponds to log2 of the fold change (log2FC) relative to Col-0 at each timepoint determined from 3 biological replicates. Wald test results for genes shown can be found in Table S1.

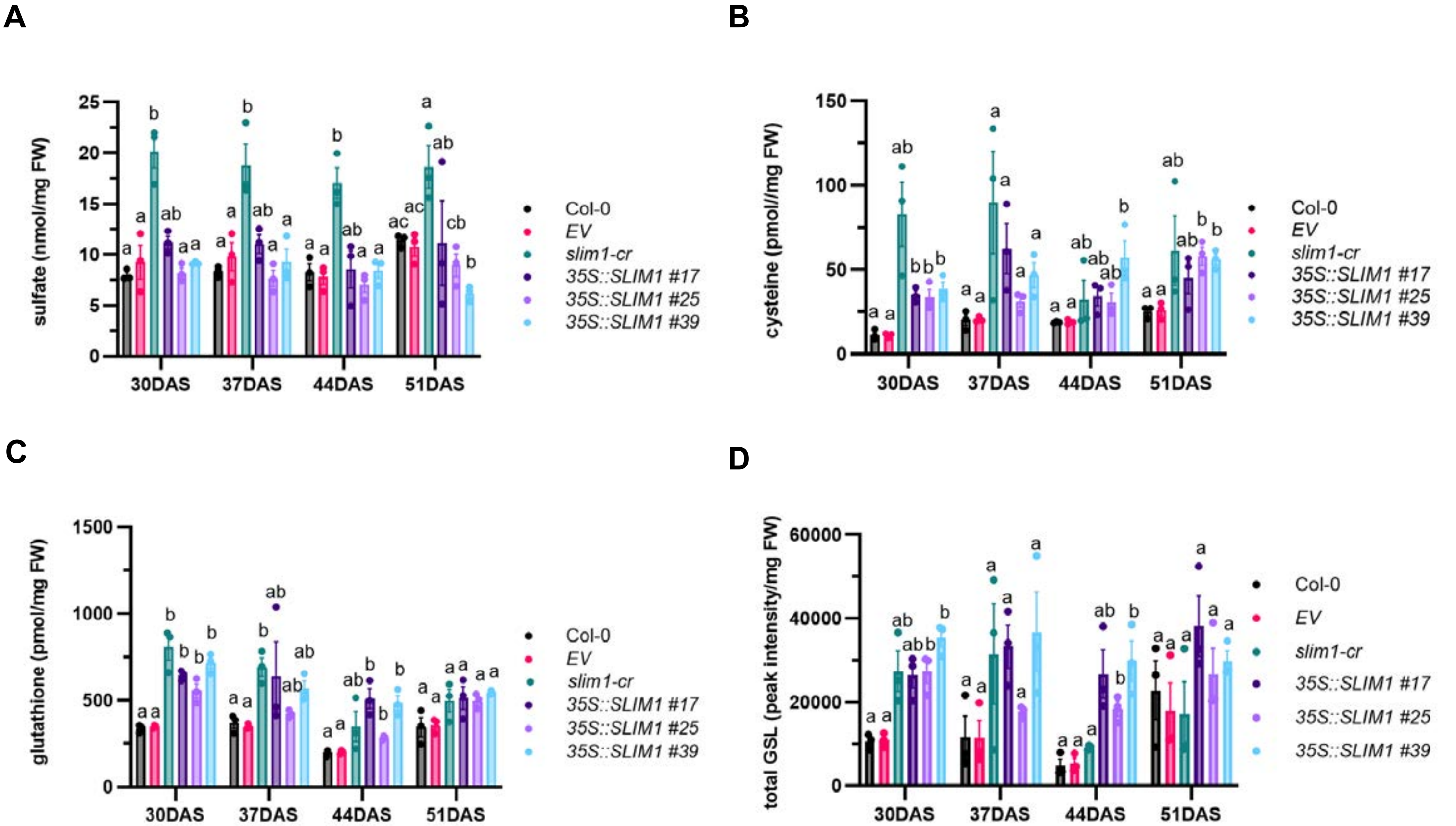

Supplementary Figure 4

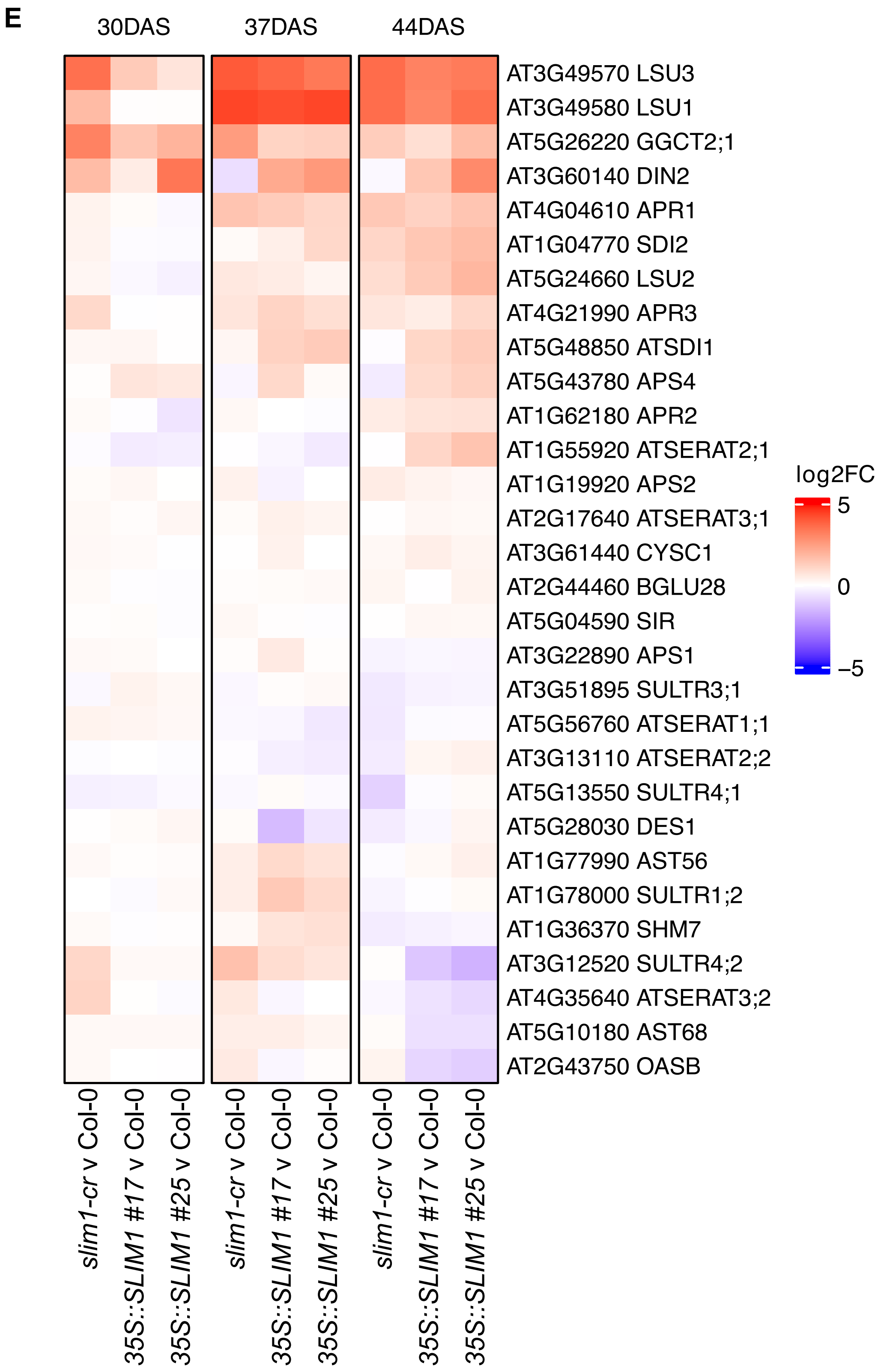

Supplementary Figure 4

F

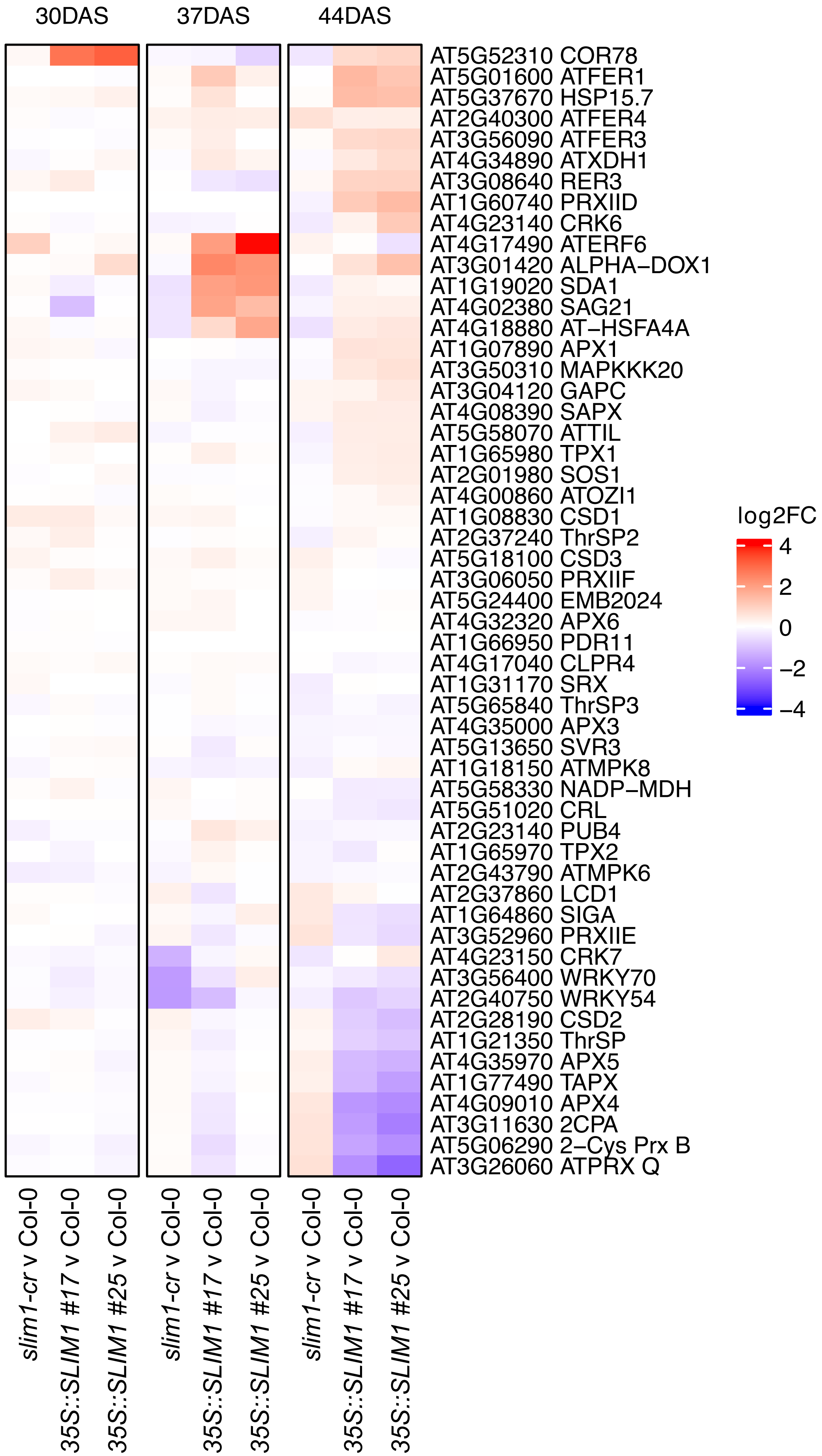

Supplement: Supplementary file 5 [file Image_2.pdf]
